# Economic growth and suicide rates: Differential accumulated effects

**DOI:** 10.1371/journal.pone.0327630

**Published:** 2025-07-07

**Authors:** Dong-Wook Lee, Yun-Chul Hong, Je-Yeon Yun, Soo-Hyun Nam, Nami Lee

**Affiliations:** 1 Department of Occupational and Environmental Medicine, Inha University Hospital, Inha University, Incheon, Republic of Korea; 2 Department of Human Systems Medicine, College of Medicine, Seoul National University, Seoul, Republic of Korea; 3 Seoul National University Hospital, Seoul, Republic of Korea; 4 Yeongeon Student Support Center, Seoul National University College of Medicine, Seoul, Republic of Korea; 5 Department of Nursing, Gyeongkuk National University, Andong, Republic of Korea; 6 Human Rights Center, Seoul National University Hospital, Seoul, Republic of Korea; 7 Public Healthcare Center, Seoul National University Hospital, Seoul, Republic of Korea; University of Pretoria, SOUTH AFRICA

## Abstract

Economic growth has a protective effect against suicide, but the nature of this association remains unclear. This ecological study explored the relationship between economic growth and suicide rates across countries within a specific timeframe. Data on age-standardized suicide rates and gross domestic product per capita (GDPpc) from 198 countries between 1991 and 2021 were obtained from the Global Burden of Disease Study and the World Bank. Using a two-way fixed-effects model and the compound annual growth rate, the association between age- and sex-adjusted suicide rates and GDPpc changes in preceding years was analyzed. GDPpc growth and lower suicide rates were significantly correlated, with a stronger correlation over longer periods, and similar associations were observed in upper-middle, lower-middle, and low-income countries. The opposite correlation was found between increased suicide rates and short-term average GDPpc growth in high-income countries, with economic growth being associated with increased suicide rates in these countries. In low- and lower-middle-income countries, increased suicide rates were associated with long-term economic stagnation. Socioenvironmental stress related to economic changes should be considered when implementing suicide prevention policies.

## Introduction

Suicide is a major preventable cause of death worldwide. In 2019, the World Health Organization (WHO) reported 703,000 deaths annually due to suicide, accounting for 1.3% of all deaths [1]. Suicide is the second leading cause of death among people aged 15–29 years [2]. While suicide may be a personal choice, exacerbating external forces, including economic instability and hazardous social systems, can contribute to it [3]. Since Durkheim suggested that socioeconomic factors are related to suicide [4], other studies have examined the associations of social, cultural, and economic factors with suicide rates at the population level [5,6]. Although over 79% of global suicides were reported in low- and middle-income countries in 2016 [7], economic hardships, including unemployment [8], housing and financial problems [9], and living in rural areas [10], could also be driving factors for suicide in higher-income countries. Studies on the relationship between socioeconomic indicators and suicide tend to depict a complex association [5,11,12].

Generally, a strong and stable national economy, represented by gross domestic product per capita (GDPpc), is a significant inverse predictor of mortality [13]. Many researchers have reported a countercyclical pattern, suggesting that recessions are associated with higher mortality due to rising unemployment, financial hardship, and reduced access to healthcare [14–17]. However, other studies indicate a procyclical relationship between economic growth and mortality, which tends to rise during economic booms, citing factors such as increased work-related stress, reduced leisure time for health-promoting activities, and higher exposure to pollution and accidents [18–25]. This inconsistency across studies may stem from the varying time horizons: short-term spikes in stress and disruptive social changes versus long-term improvements in medical infrastructure and social safety nets, as well as differences in sample composition, income levels, and other socioeconomic factors.

Regarding suicide, Ruhm and colleagues demonstrated a procyclical relationship between the economy and mortality, yet they also identified an association between economic downturns and higher suicide rates [26]. Other studies demonstrated that national economic growth indicators may negatively correlate with suicide at certain income ranges or time points [27–29]. The conflicting data could also be explained by the fact that short-term economic growth may disrupt the old socioeconomic system, increasing stress without social support, whereas long-term economic growth facilitates social safety and promotes health services [13,30]. As mental illness is bidirectional between society and individuals (involving body and mind, the environment, and genes), a single factor driving suicide cannot be determined. To the best of our knowledge, the association between suicide rates and economic growth changes in the short and long term has not been fully studied. To address this gap, instead of analyzing cross-sectional economic changes, we focused on longitudinal changes in suicide risk in various countries.

Building upon Schumpeter’s concept of “creative destruction,” rapid economic expansion does not merely lead to overall prosperity but can also displace workers, disrupt traditional social structures, and widen inequalities [31]. Such disruptions may marginalize individuals who are unable to adapt, heighten financial strain, and cause psychological stress, potentially translating into increased suicide rates. For example, short-term economic expansion can disrupt established socioeconomic structures, intensify stress, and negatively affect mental health, especially among vulnerable populations [32]. Contrarily, growth over longer periods may allow social welfare systems and healthcare infrastructure to develop and mitigate suicide risk [33]. This perspective highlights the importance of exploring the short-term disruptive effects of economic expansion and long-term protective effects of sustained growth.

Economic changes, such as GDP growth or contraction, influence employment levels, consumer sentiment, and economic stability, which affect suicide rates. Additionally, sex differences have been found in the association between suicide and economic shocks [34]. For example, after controlling for income, debt level, and other socioeconomic variables, women exhibited approximately 30% higher overall debt stress scores than men. However, women can express their feelings and emotions more freely than men, which may help protect them from suicide [35]. As the economic environment shaped by GDP growth influences employment patterns, financial stress, and social stability differently according to sex, examining these sex-specific pathways is essential for formulating targeted policies for suicide prevention in the context of changing economic conditions.

This ecological study aimed to explore the association between countries’ suicide rates and past economic growth rates using age-standardized suicide rates from 2000 to 2018 and GDPpc growth rates from 1980 to 2018. We hypothesized that 1) economic expansion may be followed by an increase in suicide rates, 2) economic growth over a long period may be associated with a decrease in suicide rates, and 3) the effect will differ according to the income level of countries and between sexes.

## Methods

### Data collection

This study used estimates from the 2021 Global Burden of Disease (GBD) study conducted by the Institute for Health Metrics and Evaluation. The GBD provides mortality and morbidity data for 204 countries and territories covering 288 causes of death [36,37]. Cause-specific mortality rates were estimated using the Cause of Death Ensemble Model, vital signs, verbal autopsy studies, partial urban vital signs, and survey/census data [38]. Age-standardized mortality rates due to self-harm, representing suicide rates for both sexes (separately and combined) in 198 countries from 1990 to 2021, were obtained from the Global Health Exchange database of the Institute for Health Metrics and Evaluation. Of the 203 countries with age-adjusted suicide rates available during the study period, we excluded 5 that could not be classified using the World Bank Atlas method (Palestine, Cook Islands, and Niue) or for which GDPpc data were not available (Democratic People’s Republic of Korea and Taiwan).

The annual GDPpc growth (%) from 1960 to 2021 (US$) was calculated from the annual GDPpc derived from World Bank open data. The average annual growth percentage of GDPpc for different periods from 1 to 20 years was calculated using the compound annual growth rate (CAGR) formula. Countries were grouped according to income level based on the data repository of gross national income per capita in June 2020, according to the World Bank Atlas method, as follows: low-income, $1,035 or less; lower-middle-income, $1,036–$4,045; upper-middle-income, $4,046–$12,535; and high-income, $12,536 or more (S1 Fig in S1 File). Data on potential confounding variables were also obtained from the World Bank data repository. Unemployment as a percentage of the total workforce (%), percentage of population over 65 years (%), and fertility rate per woman (%) were selected based on previous studies that reported a significant association between GDPpc, unemployment, female labor force, fertility rate, population over 65 years, and suicide rates at the ecological level [5,12]. Considering data availability (S1 Table in S1 File), we analyzed the period from 1991 to 2021.

### Statistical analysis

Given our 31-year repeated-measures data, a two-way fixed-effects (TWFE) model was adopted to investigate the association between annual GDP growth and suicide rates, controlling for potential confounders [39,40]. We retained the standard TWFE approach, which is commonly used in macroeconomic and ecological modeling studies. A model was constructed to assess the association between GDPpc growth and age-adjusted suicide rate:


$$Y_{jt}\;\sim \;\beta \cdot {△GDPpc}_{ky\;jt}+\gamma \cdot X_{jt}+\alpha_{j}+\;\varphi_{j}t+\varepsilon_{jt}$$

where *j* and *t* represent the index country and year, respectively; *Y*_*jt*_ is the age-standardized suicide rate for the j^th^ country at the t^th^ time; *α*_*j*_ represents country-specific fixed effects; *β* denotes the estimated effects of △GDPpc_ky_ for the j^th^ country at the t^th^ time; △GDPpc_ky_ refers to the mean annual GDPpc growth during the periods of K-1 to K (one year before, △GDPpc_1y_), K-2 to K (two years before, △GDPpc_2y_), …, and K-19 to K (20 years before, △GDPpc_20y_); γ represents the estimated effects of covariate X_jt_; X_jt_ is a vector of covariates of the j^th^ country at the t^th^ time; $\varphi_{j}$_t_ denotes time effects; and ∊_jt_ is the error term.

Considering the available timeframe of covariates and related studies, we adjusted for annual GDPpc (US$), proportion of the population aged over 65 years (%), fertility rate per female, unemployment as a proportion of the total workforce (%), and female employment (%) [5,41,42]. We estimated the strength of the association between the age-standardized annual suicide rate and average GDPpc growth in previous years. For the explanatory variable of the suicide rate, we used the average annual growth percentage of GDPpc, calculated using the CAGR formula, from the GDPpc growth of the previous year to the average value of GDPpc over the past 20 years: GDPpc growth from 1 year earlier (△GDPpc_1y_), 2 years (△GDPpc_2y_), and 20 years (△GDPpc_20y_) to the year in which the age-standardized suicide rates were calculated. The association between GDPpc_1–20y_ and the annual age-standardized suicide rate was investigated separately by income status within countries (high, upper-middle, and lower-middle to low income). Stratified analyses were performed for men and women. To assess the robustness of our results, we conducted sensitivity analyses with five progressively adjusted models: Model 1 included only GDP growth; Model 2 added the unemployment rate; Model 3 further included the proportion of the population aged over 65; Model 4 added the female labor force participation rate; and Model 5 incorporated the fertility rate in addition to all previously mentioned covariates. All statistical analyses were performed using R version 4.2.3, with the R package *plm* for TWFE models.

## Results

Table 1 shows the mean age-standardized suicide rate, GDPpc, GDPpc growth, unemployment, population over 65 years of age, female employment, and fertility rates from 1990 to 2021. The mean age-standardized suicide rate in the 198 countries (± standard deviation [SD]) was 12.5 (± 9.2) and 9.6 (± 6.3) in 1991 and 2021, respectively. The average GDPpc values were 5,826 US$ in 1991 and 17,785 US$ in 2021.

**Table 1:** Descriptive statistics for suicide rates and country-level variables.

Year	Suicide rate (per 100,000 persons)		GDPpc (US$)		GDPpc growth %		Unemployment (% total workforce)		Population over 65 years (%)		Female employment (%)		Fertility rate (births per woman)	
	Mean ± SD	N (%)	Mean ± SD	N (%)	Mean ± SD	N (%)	Mean ± SD	Mean ± SD	Mean ± SD	N (%)	Mean ± SD	N (%)	Mean ± SD	N (%)
1991	12.5 ± 9.2	198 (100)	5826 ± 9999	181 (91.4)	2.1 ± 18.6	180 (90.9)	7.6 ± 6.1	180 (90.9)	6.1 ± 4.2	198 (100)	38.7 ± 10.2	180 (90.9)	4.0 ± 1.9	191 (96.5)
1992	12.6 ± 9.3	198 (100)	6040 ± 10644	184 (92.9)	1.0 ± 17.3	181 (91.4)	8.0 ± 6.4	180 (90.9)	6.2 ± 4.3	198 (100)	38.8 ± 10.1	180 (90.9)	3.9 ± 1.8	192 (97.0)
1993	12.8 ± 9.6	198 (100)	5746 ± 9988	188 (94.9)	3.1 ± 25.4	185 (93.4)	8.4 ± 6.4	180 (90.9)	6.3 ± 4.3	198 (100)	39.0 ± 10.0	180 (90.9)	3.8 ± 1.8	191 (96.5)
1994	12.9 ± 9.8	198 (100)	6082 ± 10614	188 (94.9)	4.7 ± 27.9	188 (94.9)	8.6 ± 6.3	180 (90.9)	6.3 ± 4.4	198 (100)	39.1 ± 10	180 (90.9)	3.7 ± 1.8	191 (96.5)
1995	13.0 ± 9.8	198 (100)	6827 ± 12086	190 (96.0)	14.3 ± 22.9	188 (94.9)	8.6 ± 6.4	180 (90.9)	6.4 ± 4.4	198 (100)	39.3 ± 9.9	180 (90.9)	3.6 ± 1.8	192 (97.0)
1996	12.9 ± 9.6	198 (100)	7056 ± 12244	190 (96.0)	5.9 ± 12.9	190 (96.0)	8.8 ± 6.4	180 (90.9)	6.5 ± 4.5	198 (100)	39.4 ± 9.9	180 (90.9)	3.6 ± 1.8	191 (96.5)
1997	12.8 ± 9.4	198 (100)	6940 ± 11542	190 (96.0)	2.7 ± 14.9	190 (96.0)	8.6 ± 6.2	180 (90.9)	6.6 ± 4.6	198 (100)	39.5 ± 9.8	180 (90.9)	3.5 ± 1.8	192 (97.0)
1998	12.7 ± 9.2	198 (100)	6883 ± 11654	191 (96.5)	−0.8 ± 13.1	190 (96.0)	8.7 ± 6.2	180 (90.9)	6.6 ± 4.6	198 (100)	39.7 ± 9.7	180 (90.9)	3.4 ± 1.8	192 (97.0)
1999	12.7 ± 9.1	198 (100)	7222 ± 12144	192 (97.0)	0.9 ± 14.2	191 (96.5)	8.8 ± 6.2	180 (90.9)	6.7 ± 4.7	198 (100)	39.9 ± 9.7	180 (90.9)	3.4 ± 1.8	192 (97.0)
2000	12.6 ± 9.0	198 (100)	7222 ± 11830	193 (97.5)	4.7 ± 26.3	192 (97.0)	8.7 ± 6.3	180 (90.9)	6.8 ± 4.8	198 (100)	40.0 ± 9.6	180 (90.9)	3.3 ± 1.7	193 (97.5)
2001	12.4 ± 8.8	198 (100)	7189 ± 11800	193 (97.5)	1.1 ± 13.5	193 (97.5)	8.6 ± 6.3	180 (90.9)	6.9 ± 4.8	198 (100)	40.1 ± 9.6	180 (90.9)	3.3 ± 1.7	192 (97.0)
2002	12.2 ± 8.7	198 (100)	7843 ± 12711	197 (99.5)	5.4 ± 13.6	193 (97.5)	8.7 ± 6.3	180 (90.9)	7.0 ± 4.9	198 (100)	40.2 ± 9.6	180 (90.9)	3.2 ± 1.7	192 (97.0)
2003	12.1 ± 8.5	198 (100)	9100 ± 15010	197 (99.5)	14.1 ± 13.4	197 (99.5)	8.7 ± 6.3	180 (90.9)	7.1 ± 5.0	198 (100)	40.3 ± 9.5	180 (90.9)	3.2 ± 1.7	192 (97.0)
2004	11.9 ± 8.4	198 (100)	10392 ± 17033	197 (99.5)	15.4 ± 12.0	197 (99.5)	8.6 ± 6.2	180 (90.9)	7.1 ± 5.1	198 (100)	40.4 ± 9.5	180 (90.9)	3.1 ± 1.7	192 (97.0)
2005	11.8 ± 8.3	198 (100)	11317 ± 18188	197 (99.5)	12.6 ± 11.8	197 (99.5)	8.3 ± 6.1	180 (90.9)	7.2 ± 5.1	198 (100)	40.5 ± 9.5	180 (90.9)	3.1 ± 1.6	193 (97.5)
2006	11.5 ± 8.1	198 (100)	12355 ± 19868	197 (99.5)	13.1 ± 17.4	197 (99.5)	7.9 ± 5.9	180 (90.9)	7.3 ± 5.2	198 (100)	40.5 ± 9.4	180 (90.9)	3.1 ± 1.6	193 (97.5)
2007	11.3 ± 8.0	198 (100)	14098 ± 23173	197 (99.5)	15.9 ± 10.6	197 (99.5)	7.5 ± 5.7	180 (90.9)	7.4 ± 5.3	198 (100)	40.6 ± 9.4	180 (90.9)	3.0 ± 1.6	193 (97.5)
2008	11.2 ± 7.9	198 (100)	15416 ± 25076	198 (100)	15.3 ± 12.2	197 (99.5)	7.4 ± 5.5	180 (90.9)	7.5 ± 5.4	198 (100)	40.6 ± 9.4	180 (90.9)	3.0 ± 1.6	193 (97.5)
2009	11.2 ± 7.8	198 (100)	13669 ± 21721	198 (100)	−5.4 ± 15.4	198 (100)	8.1 ± 5.7	180 (90.9)	7.5 ± 5.4	198 (100)	40.8 ± 9.3	180 (90.9)	3.0 ± 1.5	193 (97.5)
2010	11.0 ± 7.7	198 (100)	14314 ± 21949	198 (100)	9.3 ± 11.7	198 (100)	8.3 ± 6.0	180 (90.9)	7.6 ± 5.5	198 (100)	40.9 ± 9.3	180 (90.9)	3.0 ± 1.5	193 (97.5)
2011	10.9 ± 7.6	198 (100)	15726 ± 24105	198 (100)	11.5 ± 9.40	198 (100)	8.3 ± 6.1	180 (90.9)	7.7 ± 5.6	198 (100)	40.9 ± 9.2	180 (90.9)	2.9 ± 1.5	192 (97.0)
2012	10.8 ± 7.4	198 (100)	15626 ± 23291	197 (99.5)	2.6 ± 12.0	197 (99.5)	8.3 ± 6.2	180 (90.9)	7.9 ± 5.8	198 (100)	41.0 ± 9.2	180 (90.9)	2.9 ± 1.5	194 (98.0)
2013	10.6 ± 7.3	198 (100)	16070 ± 24325	197 (99.5)	4.0 ± 9.80	197 (99.5)	8.3 ± 6.2	180 (90.9)	8.0 ± 5.9	198 (100)	41.0 ± 9.2	180 (90.9)	2.9 ± 1.4	192 (97.0)
2014	10.5 ± 7.1	198 (100)	16246 ± 24693	197 (99.5)	1.8 ± 6.40	197 (99.5)	8.1 ± 6.1	180 (90.9)	8.2 ± 6.1	198 (100)	41.1 ± 9.1	180 (90.9)	2.9 ± 1.4	192 (97.0)
2015	10.4 ± 7.0	198 (100)	14575 ± 21846	196 (99.0)	−7.6 ± 10.7	196 (99.0)	8.1 ± 5.9	180 (90.9)	8.3 ± 6.2	198 (100)	41.2 ± 9.0	180 (90.9)	2.8 ± 1.4	193 (97.5)
2016	10.3 ± 6.9	198 (100)	14756 ± 22167	195 (98.5)	−0.1 ± 9.40	195 (98.5)	7.9 ± 5.8	180 (90.9)	8.5 ± 6.4	198 (100)	41.3 ± 8.9	180 (90.9)	2.8 ± 1.4	192 (97.0)
2017	10.1 ± 6.7	198 (100)	15572 ± 22948	195 (98.5)	6.2 ± 7.60	195 (98.5)	7.7 ± 5.6	180 (90.9)	8.7 ± 6.5	198 (100)	41.3 ± 8.9	180 (90.9)	2.7 ± 1.3	192 (97.0)
2018	10.1 ± 6.6	198 (100)	16560 ± 24655	195 (98.5)	6.1 ± 9.60	195 (98.5)	7.4 ± 5.6	180 (90.9)	8.9 ± 6.6	198 (100)	41.3 ± 9.0	180 (90.9)	2.7 ± 1.3	192 (97.0)
2019	10.0 ± 6.6	198 (100)	16427 ± 24544	195 (98.5)	0.3 ± 6.50	195 (98.5)	7.2 ± 5.5	180 (90.9)	9.1 ± 6.7	198 (100)	41.4 ± 9.0	180 (90.9)	2.7 ± 1.3	192 (97.0)
2020	9.7 ± 6.5	198 (100)	15430 ± 23438	195 (98.5)	−6.7 ± 9.60	195 (98.5)	8.3 ± 5.9	180 (90.9)	9.3 ± 6.8	198 (100)	41.3 ± 8.9	180 (90.9)	2.6 ± 1.3	193 (97.5)
2021	9.6 ± 6.3	198 (100)	17785 ± 28062	193 (97.5)	12.4 ± 11.8	193 (97.5)	8.0 ± 5.9	180 (90.9)	9.5 ± 6.9	198 (100)	41.6 ± 9.0	180 (90.9)	2.6 ± 1.3	192 (97.0)

GDPpc = gross domestic product per capita; DALY = disease-adjusted life year.

**Fig 1:**
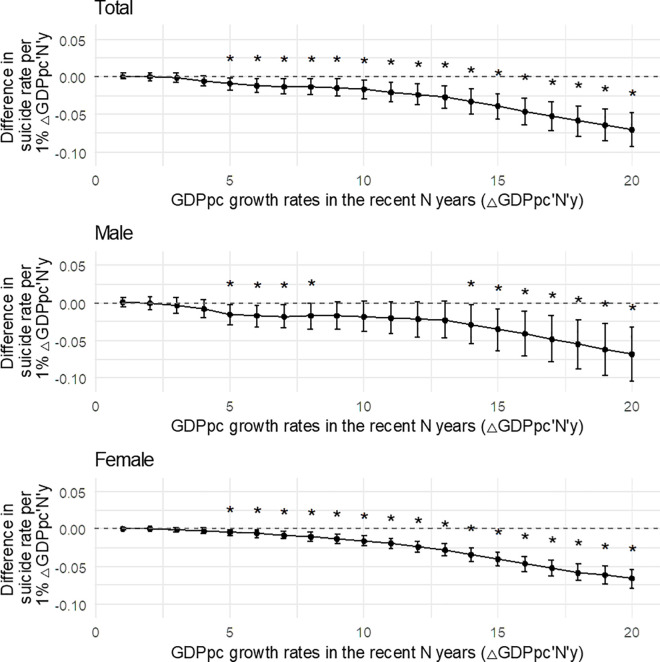
Association between average percentage change in GDPpc over different time periods (years) and age-standardized suicide rate. The x-axis shows the number of years used to calculate the average GDPpc growth from the year in which the suicide rate was recorded. Point estimates and 95% confidence intervals (CIs) show the difference in suicide rates in countries with 1% GDPpc growth during a specific period. For example, in “Both sexes,” point estimates and 95% CIs show how an average GDPpc growth over three years is associated with the suicide rate in the last year of those three years. * p < 0.05.

Fig 1 and S2 Table in S1 File show the extent of the association and its confidence intervals (CIs) between age-standardized suicide rates (per 100,000 persons) and average GDPpc growth over the specified period after adjusting for annual GDPpc, population aged > 65 years, fertility rate, unemployment, and women’s employment. In the long term, higher GDPpc growth rates were significantly associated with lower suicide rates, with a more pronounced association with the GDPpc growth rate using the CAGR. At the country level, a 1-percentage-point increase in GDPpc_20y_ was significantly associated with lower suicide rates: −0.0703 (95% CI = −0.0925–-0.0481), −0.068 (95% CI = −0.1036–-0.0325), and −0.0656 (95% CI = −0.0783–-0.0528) per 100,000 people for both sexes, men, and women. In contrast, GDPpc growth rates in recent years (△GDPpc_1y_, △GDPpc_2y_, △GDPpc_3y_, and △GDPpc_4y_) were not significantly associated with suicide rates. The numbers of observations, degrees of freedom, R-squared values, and F-values of the statistical models are listed in S3 Table in S1 File.

**Fig 2:**
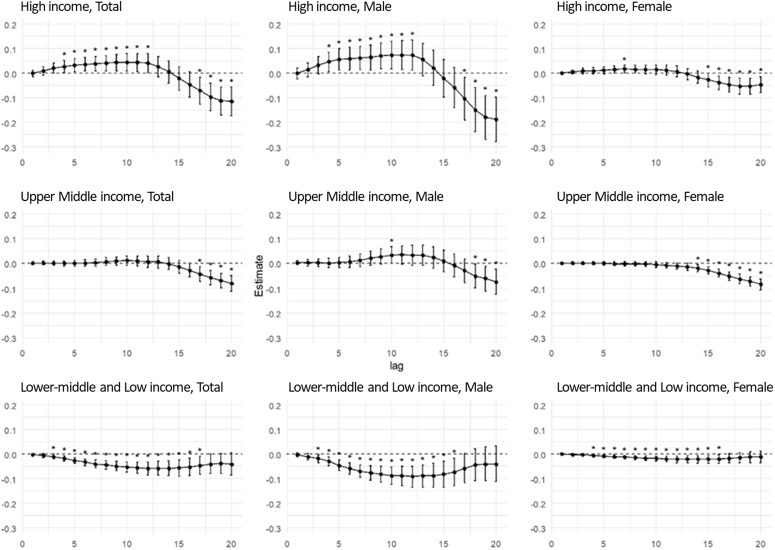
Association between average percentage change in GDPpc over different time periods (years) and age-standardized suicide rate by countries according to income. The x-axis shows the number of years used to calculate the average GDPpc growth from the year in which the suicide rate was recorded. Point estimates and 95% CIs show the difference in suicide rates in countries with a 1% growth in GDPpc during a specific period. For example, in “Both sexes,” point estimates and 95% CIs show how an average growth of GDPpc over three years is associated with the suicide rate in the last year of those three years. All models were adjusted for annual GDPpc (US$), proportion of the population aged over 65 years (%), fertility rate per woman, unemployment as a proportion of the total workforce (%), and women’s employment (%). The income groups of the countries were classified based on the World Bank Atlas method using the gross national income per capita in June 2020, as follows: low-income, $1,035 or less; lower-middle-income, $1,036–4,045; upper-middle income, $4,046–12,535; and high-income, $12,536 or more. * p < 0.05.

Fig 2 and S4 Table in S1 File present the results of the stratified analyses according to the income level of each country. In the high-income group, the associations between △GDPpc_4y_ – △GDPpc_12y_ and higher suicide rates and △GDPpc_17y_ – △GDPpc_20y_ and lower suicide rates were significant. In men, a significant positive correlation was observed between the increase in GDPpc (△GDPpc_4y_ – △GDPpc_14y_) and higher suicide rates. In women, △GDPpc_7y_ was associated with lower suicide rates in high-income countries. Long-term GDPpc growth was significantly associated with lower suicide rates in men and women in high-income countries. In upper-middle-income countries, no significant association was observed in the short term, except for that between △GDPpc_10y_ and higher suicide rates in men. △GDPpc_17y_ – △GDPpc_20y_ was associated with lower suicide rates, implying that long-term economic growth was associated with lower suicide rates. In lower-middle- and low-income countries, negative associations between GDPpc growth and suicide rates began to emerge. Significant reductions in suicide rates were observed in both sexes and the total population by the fifth year, with stronger long-term effects. The numbers of observations, degrees of freedom, R-squared values, and F-values of the statistical models are listed in S5 Table in S1 File. The coefficients, standard errors, and p-values of the variables included in the constructed models are presented in the Supplementary File 1. Additionally, as shown in S2 Fig in S1 File, these observations were robust across various models without substantial differences.

## Discussion

At the country level, accumulated GDPpc growth was significantly associated with a decrease in suicide rates, with a stronger association over longer periods. However, short-term economic growth in high-income countries was significantly associated with higher suicide rates, with a more pronounced effect in men than in women. To the best of our knowledge, this is the first ecological study to investigate the differential effects of short- and long-term economic changes on suicide by comparing phase differences according to countries’ income levels.

We found a potential effect of long-term economic growth on suicide rates, especially in relatively lower-income countries. The association between economic hardship and suicide rates has been extensively studied. Hilario et al. investigated the association between suicide rates and GDPpc for the same year in 56 countries (from 1980 to 2007) using the WHO mortality and World Bank databases. The association between purchasing power parity-adjusted GDPpc and suicide rates was positive in Latin American, Caribbean, Southeast Asian, and high-income Asian countries but not in European countries, Canada, Australia, New Zealand, or Africa [43]. In Shangdong, China, a decrease in the suicide rate was correlated with economic growth from 1982 to 2005 [44]. In Malaysia, suicide rates declined between 2000 and 2013 but increased between 2014 and 2019, showing a pattern similar to that of the country’s economic status during this period [45].

Currently, approximately 75% of suicides worldwide occur in low- and middle-income countries with high poverty rates [46]. Long-term low GDP growth in these countries may contribute to increased suicide rates through economic and psychological mechanisms more explicitly than in high-income countries. First, financial strain may increase an individual’s risk of suicide or indirectly exacerbate existing physical and/or mental illnesses, which may be more difficult to manage in low-income countries. Second, low- and middle-income countries may lack sufficient budgets to establish social safety nets to mitigate economic adversity. Third, psychologically, long-term economic stagnation can make people suffering from financial strain feel hopeless, reinforcing the feeling of being trapped with no escape [47].

Contrarily, a positive correlation between the economic growth index and suicide rates has also been reported. As low economic growth can affect suicide rates through various pathways, these complex findings across studies should be carefully interpreted. Milner et al. studied effects similar to those studied by Hilario et al. but focused on various socioeconomic determinants and found that higher unemployment rates, a greater proportion of women in the labor force, lower health expenditure, a larger population aged over 65 years, and a lower fertility rate were associated with a country’s suicide rate [5].

We found a significant relationship between short-term economic growth in high-income countries and higher suicide rates. Granados reported increasing mortality rates during the US economic expansion between 1900 and 1996. The different components of economic growth explain the changes in all-cause mortality. A year-to-year change in GDPpc was associated with an increase in mortality, referred to as an “oscillation,” whereas there was a long-term trend of increasing GDPpc associated with decreasing all-cause mortality [48]. This study found that suicide rates generally declined with economic expansion and increased with recession. However, in our study, the recent economic expansion was associated with increased suicide rates in high- and upper-middle-income countries. Instead of concluding with a simple correlation between GDP and suicide rates, the cumulative effects of socioeconomic growth, considering confounding variables, should be carefully examined. In the United States, suicide mortality rates increased pervasively in 1999 and 2017, with lower manufacturing employment, higher opioid prescriptions, and increased gun accessibility for men as contextual predictors [49]. Although debates on cultural differences were not included in this article, speculating the association between rapidly changing cultural norms from hierarchical and male-centered to democratic and gender-neutral cultures in developing countries is possible.

One explanation for this association is that the negative effects of income inequality derived from economic growth exceed the positive effects of economic growth. Economic expansion may be unavoidable in the pursuit of a better society; however, rapid economic development without a proper social safety net and healthcare system often leads to unpredictable social upheaval and mental distress [13,48]. Kuznets’ inverted U-curve hypothesis argues that income inequality improves in advanced countries when they grow over the long term and reach a stabilization phase [50]. However, recent claims indicate that income inequality can worsen even with economic growth. Deininger and Squire argue that income inequality and long-term economic growth are positively correlated [51]. Benabou et al. argue that economic growth and income inequality have a positive relationship because the benefits of economic growth are monopolized by the middle- and high-income classes [52]. A study using data from 5,507 Brazilian municipalities from 2000 to 2011 found that income inequality was a community-level risk factor for suicide, which partially supports this explanation [53].

Creative destruction accompanied by economic growth, especially in high-income countries, triggers mental stress among individuals. The speed and intensity of economic development, accompanied by new workloads and overconsumption, may become overwhelming and intolerable in an accelerated growth period compared with a gradual expansion period. A lagging labor supply and an unstable job market may fail to provide sufficient time for workers to learn, train, and adapt to new operational systems [54]. Thus, rapid economic growth and suicide may be linked through the social stress caused by economic uncertainty. According to Claveria et al., the association between suicide rates and economic uncertainty, calculated using the Economic Policy Uncertainty Index, was reported using a panel model for 183 countries between 2000 and 2019 [55].

Another possible explanation is that the race for economic growth may have a spillover effect on mental health. Analyzing WHO data from 73 countries from 1990 to 2010, a suicidal Kuznets curve showed that the suicide rate decreased during economic growth in certain sections but increased after exceeding a certain income level [29]. This shows that insecurity and unpredictable social changes can be strong stressors, leading to increased suicide rates.

In our study, short-term GDPpc growth was more strongly associated with suicide rates in men than in women. Suicide rate trends in England and Wales before and after the 2008 economic recession showed that economic strain and unemployment increased suicide rates among middle-aged men [56]. Berk et al. also reported that male suicide rates were significantly associated with an increase in economic adversity markers; however, the opposite pattern was found in women [57]. Similarly, in our study, the association between GDPpc growth and suicide rates was stronger among men than women.

This could be explained by individual preferred competency change in the job market and related job loss. Rapid economic growth is usually accompanied by technological progress, which can lead to job losses [58]. As financial and economic issues are major sources of stress [59], unemployment, job insecurity, and financial losses can be individual suicide risk factors. Furthermore, usually men are the breadwinners, particularly in lower- and middle-income countries; thus, they experience greater shame regarding unemployment [60] and tend to use violence to solve hardships in the absence of social or family safety nets.

This study highlights the need for targeted interventions to address the potential rise in suicide rates during periods of economic expansion. Multiple evidence suggest that certain policy measures can significantly reduce population-level suicide. For example, in Finland, community-based mental health services showed a beneficial effect on suicide [61]. A cross-national study also showed that national suicide prevention programs can decrease suicide rates [62]. Further, a time-series study across 30 countries demonstrated the suicide prevention effects of economic welfare safety nets [63]. Together, these findings underline the critical role that robust, context-specific policies can play in mitigating the adverse impacts of economic growth on suicide.

Our study had several limitations. First, the ecological study design may have led to a generalized fallacy in the interpretation. Additionally, because economic growth and suicide are interactively correlated, a causal conclusion cannot be drawn solely by observing the associations between earlier economic indicators and subsequent mortality rates. This constitutes a key limitation of our study. Second, we used indices derived from GBD study estimates, which have inherent limitations in terms of the reliability and validity of the data. In low- and middle-income countries, mental illness and suicide may be underreported owing to social stigma or financial constraints. A recent meta-analysis on the stigmatization of mental illness suggested that psychiatric illness and suicide in middle- and low-income countries may be underreported [64]. Inadequate public data on suicide and mental illness owing to ineffective death registration should also be considered in low-income countries [65]. Changes in economic conditions may indirectly affect the quality of suicide data over time. Periods of economic growth can lead to better-resourced public health systems, resulting in more accurate and complete suicide reporting. However, an economic recession may strain public health resources, leading to under-reporting. Third, our study did not explore other social risk factors related to suicide, including community safety, acculturation-related stress (e.g., among indigenous or displaced persons), discrimination, sense of isolation, abuse, and violence. Other medical factors, including alcohol abuse and chronic pain [7], have not been investigated in relation to suicide or their association with GDP. We could not analyze the relationship between suicide rates and distinct social factors such as unemployment, population aging, healthcare expenditure, and fertility rates, which requires further investigation. Finally, a methodological limitation arose from the use of the traditional TWFE model. Methodological advancements now address TWFE models with continuous treatments, offering insights into their causal interpretation [66]. Future research could therefore benefit from exploring more appropriate approaches for analyzing continuous variables without sacrificing critical information.

## Conclusions

Economic growth affects suicide rates. Balanced rapid expansion may lead to social disruption in high-income countries, whereas consistent long-term growth appears to protect against suicide. In high-income countries, suicide rates are higher in the early rather than later phases of economic growth, and this phenomenon is more clearly observed in men than in women. In low- and lower-middle-income countries, steady economic development is crucial for alleviating poverty, which is a significant contributor to suicide risk. Our study suggests that the early phase of rapid economic development can create a stressful environment, which in turn contributes to increased suicide rates once a country reaches high-income status. Coupled with social change, a country’s economic development requires a more comprehensive strategic governmental approach to address the consequences for mental healthcare. Future research is needed to mitigate the detrimental effects of long-term economic stagnation in low- and middle-income countries and the adverse effects of rapid economic growth in high-income countries through interventions or policy programs.

## Supporting information

S1 File Supporting information for the article: Included countries and Data availability across the included countries (S1 Table), Associations between the average GDPpc growth in different time window (years) and age-standardized suicide rate (S2 Table), Diagnostics and number of observations for the associations between the average of absolute values of % change of GDPpc in different time window (years) and age-standardised suicide rate (S3 Table), Associations between the average GDPpc growth in different time window (years) and age-standardised suicide rate (S4 Table), Diagnostics and number of observations for the associations between the average GDPpc growth in different time window (years) and age-standardised suicide rate (S5 Table), World Map of Countries by Income Group (S1 Fig), and Association between the average percentage change in GDPpc in different time periods (years) and age-standardized suicide rate by countries according to income in various model specifications (S2 Fig). (DOCX)

S2 File The coefficients, standard errors, and p-values of the variables included in the constructed models. (XLSX)

S3 File The data used in the analysis. (CSV)

S4 File The R code used in the analysis. (R)
