# Supplementary material for: Economic growth and suicide rates: Differential accumulated effects
Source: PLoS One. 2025 Jul 7;20(7):e0327630. doi: 10.1371/journal.pone.0327630 (PMC12233259; doi:10.1371/journal.pone.0327630)
Supplement: S1 File — (DOCX) [file pone.0327630.s001.docx]

**supplementary materials**

**Economic growth and suicide rates:
Differential accumulated effects**

Dong-Wook Lee, MD, MSc,^1^ Yun-Chul Hong, MD, PhD,^2^ Je-Yeon Yun, MD, PhD,^3,4^ Soo-Hyun Nam, MSc,^5^ and Nami Lee, MD, PhD^2,5,6*^

^1^ Department of Occupational and Environmental Medicine, Inha University Hospital, Inha University, Incheon, Republic of Korea

^2^ Department of Human Systems Medicine, College of Medicine, Seoul National University, Seoul,

Republic of Korea

^3^ Seoul National University Hospital, Seoul, Republic of Korea

^4^ Yeongeon Student Support Center, Seoul National University College of Medicine, Seoul, Republic of Korea

^5^ Human Rights Center, Seoul National University Hospital, Seoul, Republic of Korea

^6^ Public Healthcare Center, Seoul National University Hospital, Seoul, Republic of Korea

* Corresponding author

E-mail: [nami6107@naver.com](mailto:nami6107@naver.com) (NL)

**Table of contents**

[S1 Table. Included countries and Data availability across the included countries. 3](#_Toc193978530)

[S2 Table. Associations between the average GDPpc growth in different time window (years) and age-standardised suicide rate 8](#_Toc193978531)

[S3 Table. Diagnostics and number of observations for the associations between the average of absolute values of % change of GDPpc in different time window (years) and age-standardised suicide rate. 9](#_Toc193978532)

[S4 Table. Associations between the average GDPpc growth in different time window (years) and age-standardised suicide rate 10](#_Toc193978533)

[S5 Table. Diagnostics and number of observations for the associations between the average GDPpc growth in different time window (years) and age-standardised suicide rate 11](#_Toc193978534)

S1 Fig. World Map of Countries by Income Group 13

S2 Fig. Association between the average percentage change in GDPpc in different time periods (years) and age-standardized suicide rate by countries according to income in various model specifications. 14

# S1 Table. Included countries and Data availability across the included countries.

|  |  |  |  |  | **Data availability (years)** | | | | | |
| --- | --- | --- | --- | --- | --- | --- | --- | --- | --- | --- |
| **No** | **Country** | **Code** | **Region** | **Income Group** | **Suicide rate** | **GDP per capita** | **Unemployment (%)** | **Population over 65 years** | **Female employment (%) labor market** | **Fertility rate (births per woman)** |
| 1 | Afghanistan | AFG | South Asia | Low | 1980–2021 | 2000-2022 | 1991–2021 | 1980–2021 | 1991–2021 | 1980–2021 |
| 2 | Angola | AGO | Sub-Saharan Africa | Lower middle | 1980–2021 | 1993-2023 | 1991–2021 | 1980–2021 | 1991–2021 | 1980–2021 |
| 3 | Albania | ALB | Europe & Central Asia | Upper middle | 1980–2021 | 1984-2023 | 1991–2021 | 1980–2021 | 1991–2021 | 1980–2021 |
| 4 | Andorra | AND | Europe & Central Asia | High | 1980–2021 | 1970-2023 | not available | 1980–2021 | not available | 2006–2010 |
| 5 | United Arab Emirates | ARE | Middle East & North Africa | High | 1980–2021 | 1970-2023 | 1991–2021 | 1980–2021 | 1991–2021 | 1980–2021 |
| 6 | Argentina | ARG | Latin America & Caribbean | Upper middle | 1980–2021 | 1983-2023 | 1991–2021 | 1980–2021 | 1991–2021 | 1980–2021 |
| 7 | Armenia | ARM | Europe & Central Asia | Upper middle | 1980–2021 | 1990-2023 | 1991–2021 | 1980–2021 | 1991–2021 | 1980–2021 |
| 8 | American Samoa | ASM | East Asia & Pacific | Upper middle | 1980–2021 | 2002-2022 | not available | 1980–2021 | not available | not available |
| 9 | Antigua and Barbuda | ATG | Latin America & Caribbean | High | 1980–2021 | 1977-2023 | not available | 1980–2021 | not available | 1980–2021 |
| 10 | Australia | AUS | East Asia & Pacific | High | 1980–2021 | 1960-2023 | 1991–2021 | 1980–2021 | 1991–2021 | 1980–2021 |
| 11 | Austria | AUT | Europe & Central Asia | High | 1980–2021 | 1960-2023 | 1991–2021 | 1980–2021 | 1991–2021 | 1980–2021 |
| 12 | Azerbaijan | AZE | Europe & Central Asia | Upper middle | 1980–2021 | 1990-2023 | 1991–2021 | 1980–2021 | 1991–2021 | 1980–2021 |
| 13 | Burundi | BDI | Sub-Saharan Africa | Low | 1980–2021 | 1960-2023 | 1991–2021 | 1980–2021 | 1991–2021 | 1980–2021 |
| 14 | Belgium | BEL | Europe & Central Asia | High | 1980–2021 | 1960-2023 | 1991–2021 | 1980–2021 | 1991–2021 | 1980–2021 |
| 15 | Benin | BEN | Sub-Saharan Africa | Lower middle | 1980–2021 | 1960-2023 | 1991–2021 | 1980–2021 | 1991–2021 | 1980–2021 |
| 16 | Burkina Faso | BFA | Sub-Saharan Africa | Low | 1980–2021 | 1960-2023 | 1991–2021 | 1980–2021 | 1991–2021 | 1980–2021 |
| 17 | Bangladesh | BGD | South Asia | Lower middle | 1980–2021 | 1960-2023 | 1991–2021 | 1980–2021 | 1991–2021 | 1980–2021 |
| 18 | Bulgaria | BGR | Europe & Central Asia | Upper middle | 1980–2021 | 1980-2023 | 1991–2021 | 1980–2021 | 1991–2021 | 1980–2021 |
| 19 | Bahrain | BHR | Middle East & North Africa | High | 1980–2021 | 1970-2023 | 1991–2021 | 1980–2021 | 1991–2021 | 1980–2021 |
| 20 | Bahamas, The | BHS | Latin America & Caribbean | High | 1980–2021 | 1960-2023 | 1991–2021 | 1980–2021 | 1991–2021 | 1980–2021 |
| 21 | Bosnia and Herzegovina | BIH | Europe & Central Asia | Upper middle | 1980–2021 | 1990-2023 | 1991–2021 | 1980–2021 | 1991–2021 | 1980–2021 |
| 22 | Belarus | BLR | Europe & Central Asia | Upper middle | 1980–2021 | 1992-2023 | 1991–2021 | 1980–2021 | 1991–2021 | 1980–2021 |
| 23 | Belize | BLZ | Latin America & Caribbean | Upper middle | 1980–2021 | 1960-2023 | 1991–2021 | 1980–2021 | 1991–2021 | 1980–2021 |
| 24 | Bermuda | BMU | North America | High | 1980–2021 | 1960-2022 | not available | 1980–2021 | not available | 1980–2021 |
| 25 | Bolivia | BOL | Latin America & Caribbean | Lower middle | 1980–2021 | 1960-2023 | 1991–2021 | 1980–2021 | 1991–2021 | 1980–2021 |
| 26 | Brazil | BRA | Latin America & Caribbean | Upper middle | 1980–2021 | 1989-2023 | 1991–2021 | 1980–2021 | 1991–2021 | 1980–2021 |
| 27 | Barbados | BRB | Latin America & Caribbean | High | 1980–2021 | 1960-2023 | 1991–2021 | 1980–2021 | 1991–2021 | 1980–2021 |
| 28 | Brunei Darussalam | BRN | East Asia & Pacific | High | 1980–2021 | 1965-2023 | 1991–2021 | 1980–2021 | 1991–2021 | 1980–2021 |
| 29 | Bhutan | BTN | South Asia | Lower middle | 1980–2021 | 1970-2022 | 1991–2021 | 1980–2021 | 1991–2021 | 1980–2021 |
| 30 | Botswana | BWA | Sub-Saharan Africa | Upper middle | 1980–2021 | 1960-2023 | 1991–2021 | 1980–2021 | 1991–2021 | 1980–2021 |
| 31 | Central African Republic | CAF | Sub-Saharan Africa | Low | 1980–2021 | 1960-2023 | 1991–2021 | 1980–2021 | 1991–2021 | 1980–2021 |
| 32 | Canada | CAN | North America | High | 1980–2021 | 1960-2023 | 1991–2021 | 1980–2021 | 1991–2021 | 1980–2021 |
| 33 | Switzerland | CHE | Europe & Central Asia | High | 1980–2021 | 1960-2023 | 1991–2021 | 1980–2021 | 1991–2021 | 1980–2021 |
| 34 | Chile | CHL | Latin America & Caribbean | High | 1980–2021 | 1960-2023 | 1991–2021 | 1980–2021 | 1991–2021 | 1980–2021 |
| 35 | China | CHN | East Asia & Pacific | Upper middle | 1980–2021 | 1960-2023 | 1991–2021 | 1980–2021 | 1991–2021 | 1980–2021 |
| 36 | Côte d'Ivoire | CIV | Sub-Saharan Africa | Lower middle | 1980–2021 | 1960-2023 | 1991–2021 | 1980–2021 | 1991–2021 | 1980–2021 |
| 37 | Cameroon | CMR | Sub-Saharan Africa | Lower middle | 1980–2021 | 1960-2023 | 1991–2021 | 1980–2021 | 1991–2021 | 1980–2021 |
| 38 | Congo, Dem. Rep. | COD | Sub-Saharan Africa | Low | 1980–2021 | 1992-2023 | 1991–2021 | 1980–2021 | 1991–2021 | 1980–2021 |
| 39 | Congo, Rep. | COG | Sub-Saharan Africa | Lower middle | 1980–2021 | 1960-2023 | 1991–2021 | 1980–2021 | 1991–2021 | 1980–2021 |
| 40 | Colombia | COL | Latin America & Caribbean | Upper middle | 1980–2021 | 1960-2023 | 1991–2021 | 1980–2021 | 1991–2021 | 1980–2021 |
| 41 | Comoros | COM | Sub-Saharan Africa | Lower middle | 1980–2021 | 1980-2023 | 1991–2021 | 1980–2021 | 1991–2021 | 1980–2021 |
| 42 | Cabo Verde | CPV | Sub-Saharan Africa | Lower middle | 1980–2021 | 1980-2023 | 1991–2021 | 1980–2021 | 1991–2021 | 1980–2021 |
| 43 | Costa Rica | CRI | Latin America & Caribbean | Upper middle | 1980–2021 | 1960-2023 | 1991–2021 | 1980–2021 | 1991–2021 | 1980–2021 |
| 44 | Cuba | CUB | Latin America & Caribbean | Upper middle | 1980–2021 | 1970-2020 | 1991–2021 | 1980–2021 | 1991–2021 | 1980–2021 |
| 45 | Cyprus | CYP | Europe & Central Asia | High | 1980–2021 | 1975-2023 | 1991–2021 | 1980–2021 | 1991–2021 | 1980–2021 |
| 46 | Czech Republic | CZE | Europe & Central Asia | High | 1980–2021 | 1990-2023 | 1991–2021 | 1980–2021 | 1991–2021 | 1980–2021 |
| 47 | Germany | DEU | Europe & Central Asia | High | 1980–2021 | 1960-2023 | 1991–2021 | 1980–2021 | 1991–2021 | 1980–2021 |
| 48 | Djibouti | DJI | Middle East & North Africa | Lower middle | 1980–2021 | 1985-2023 | 1991–2021 | 1980–2021 | 1991–2021 | 1980–2021 |
| 49 | Dominica | DMA | Latin America & Caribbean | Upper middle | 1980–2021 | 1977-2023 | not available | 1980–2021 | not available | 1980–2021 |
| 50 | Denmark | DNK | Europe & Central Asia | High | 1980–2021 | 1960-2023 | 1991–2021 | 1980–2021 | 1991–2021 | 1980–2021 |
| 51 | Dominican Republic | DOM | Latin America & Caribbean | Upper middle | 1980–2021 | 1960-2023 | 1991–2021 | 1980–2021 | 1991–2021 | 1980–2021 |
| 52 | Algeria | DZA | Middle East & North Africa | Lower middle | 1980–2021 | 1960-2023 | 1991–2021 | 1980–2021 | 1991–2021 | 1980–2021 |
| 53 | Ecuador | ECU | Latin America & Caribbean | Upper middle | 1980–2021 | 1960-2023 | 1991–2021 | 1980–2021 | 1991–2021 | 1980–2021 |
| 54 | Egypt, Arab Rep. | EGY | Middle East & North Africa | Lower middle | 1980–2021 | 1965-2023 | 1991–2021 | 1980–2021 | 1991–2021 | 1980–2021 |
| 55 | Eritrea | ERI | Sub-Saharan Africa | Low | 1980–2021 | 1992-2011 | 1991–2021 | 1980–2021 | 1991–2021 | 1980–2021 |
| 56 | Spain | ESP | Europe & Central Asia | High | 1980–2021 | 1960-2023 | 1991–2021 | 1980–2021 | 1991–2021 | 1980–2021 |
| 57 | Estonia | EST | Europe & Central Asia | High | 1980–2021 | 1993-2023 | 1991–2021 | 1980–2021 | 1991–2021 | 1980–2021 |
| 58 | Ethiopia | ETH | Sub-Saharan Africa | Low | 1980–2021 | 1960-2023 | 1991–2021 | 1980–2021 | 1991–2021 | 1980–2021 |
| 59 | Finland | FIN | Europe & Central Asia | High | 1980–2021 | 1960-2023 | 1991–2021 | 1980–2021 | 1991–2021 | 1980–2021 |
| 60 | Fiji | FJI | East Asia & Pacific | Upper middle | 1980–2021 | 1960-2023 | 1991–2021 | 1980–2021 | 1991–2021 | 1980–2021 |
| 61 | France | FRA | Europe & Central Asia | High | 1980–2021 | 1960-2023 | 1991–2021 | 1980–2021 | 1991–2021 | 1980–2021 |
| 62 | Micronesia, Fed. Sts. | FSM | East Asia & Pacific | Lower middle | 1980–2021 | 1970-2023 | not available | 1980–2021 | not available | 1980–2021 |
| 63 | Gabon | GAB | Sub-Saharan Africa | Upper middle | 1980–2021 | 1960-2023 | 1991–2021 | 1980–2021 | 1991–2021 | 1980–2021 |
| 64 | United Kingdom | GBR | Europe & Central Asia | High | 1980–2021 | 1960-2023 | 1991–2021 | 1980–2021 | 1991–2021 | 1980–2021 |
| 65 | Georgia | GEO | Europe & Central Asia | Upper middle | 1980–2021 | 1990-2023 | 1991–2021 | 1980–2021 | 1991–2021 | 1980–2021 |
| 66 | Ghana | GHA | Sub-Saharan Africa | Lower middle | 1980–2021 | 1960-2023 | 1991–2021 | 1980–2021 | 1991–2021 | 1980–2021 |
| 67 | Guinea | GIN | Sub-Saharan Africa | Low | 1980–2021 | 1970-2023 | 1991–2021 | 1980–2021 | 1991–2021 | 1980–2021 |
| 68 | Gambia, The | GMB | Sub-Saharan Africa | Low | 1980–2021 | 1966-2023 | 1991–2021 | 1980–2021 | 1991–2021 | 1980–2021 |
| 69 | Guinea-Bissau | GNB | Sub-Saharan Africa | Low | 1980–2021 | 1970-2023 | 1991–2021 | 1980–2021 | 1991–2021 | 1980–2021 |
| 70 | Equatorial Guinea | GNQ | Sub-Saharan Africa | Upper middle | 1980–2021 | 1962-2023 | 1991–2021 | 1980–2021 | 1991–2021 | 1980–2021 |
| 71 | Greece | GRC | Europe & Central Asia | High | 1980–2021 | 1960-2023 | 1991–2021 | 1980–2021 | 1991–2021 | 1980–2021 |
| 72 | Grenada | GRD | Latin America & Caribbean | Upper middle | 1980–2021 | 1977-2023 | not available | 1980–2021 | not available | 1980–2021 |
| 73 | Greenland | GRL | Europe & Central Asia | High | 1980–2021 | 1970-2021 | not available | 1980–2021 | not available | 1980–2021 |
| 74 | Guatemala | GTM | Latin America & Caribbean | Upper middle | 1980–2021 | 1960-2023 | 1991–2021 | 1980–2021 | 1991–2021 | 1980–2021 |
| 75 | Guam | GUM | East Asia & Pacific | High | 1980–2021 | 2002-2022 | 1991–2021 | 1980–2021 | 1991–2021 | 1980–2021 |
| 76 | Guyana | GUY | Latin America & Caribbean | Upper middle | 1980–2021 | 1960-2023 | 1991–2021 | 1980–2021 | 1991–2021 | 1980–2021 |
| 77 | Honduras | HND | Latin America & Caribbean | Lower middle | 1980–2021 | 1960-2023 | 1991–2021 | 1980–2021 | 1991–2021 | 1980–2021 |
| 78 | Croatia | HRV | Europe & Central Asia | High | 1980–2021 | 1990-2023 | 1991–2021 | 1980–2021 | 1991–2021 | 1980–2021 |
| 79 | Haiti | HTI | Latin America & Caribbean | Low | 1980–2021 | 1960-2023 | 1991–2021 | 1980–2021 | 1991–2021 | 1980–2021 |
| 80 | Hungary | HUN | Europe & Central Asia | High | 1980–2021 | 1968-2023 | 1991–2021 | 1980–2021 | 1991–2021 | 1980–2021 |
| 81 | Indonesia | IDN | East Asia & Pacific | Upper middle | 1980–2021 | 1967-2023 | 1991–2021 | 1980–2021 | 1991–2021 | 1980–2021 |
| 82 | India | IND | South Asia | Lower middle | 1980–2021 | 1960-2023 | 1991–2021 | 1980–2021 | 1991–2021 | 1980–2021 |
| 83 | Ireland | IRL | Europe & Central Asia | High | 1980–2021 | 1960-2023 | 1991–2021 | 1980–2021 | 1991–2021 | 1980–2021 |
| 84 | Iran, Islamic Rep. | IRN | Middle East & North Africa | Upper middle | 1980–2021 | 1960-2023 | 1991–2021 | 1980–2021 | 1991–2021 | 1980–2021 |
| 85 | Iraq | IRQ | Middle East & North Africa | Upper middle | 1980–2021 | 1960-2023 | 1991–2021 | 1980–2021 | 1991–2021 | 1980–2021 |
| 86 | Iceland | ISL | Europe & Central Asia | High | 1980–2021 | 1960-2023 | 1991–2021 | 1980–2021 | 1991–2021 | 1980–2021 |
| 87 | Israel | ISR | Middle East & North Africa | High | 1980–2021 | 1960-2023 | 1991–2021 | 1980–2021 | 1991–2021 | 1980–2021 |
| 88 | Italy | ITA | Europe & Central Asia | High | 1980–2021 | 1960-2023 | 1991–2021 | 1980–2021 | 1991–2021 | 1980–2021 |
| 89 | Jamaica | JAM | Latin America & Caribbean | Upper middle | 1980–2021 | 1960-2023 | 1991–2021 | 1980–2021 | 1991–2021 | 1980–2021 |
| 90 | Jordan | JOR | Middle East & North Africa | Upper middle | 1980–2021 | 1965-2023 | 1991–2021 | 1980–2021 | 1991–2021 | 1980–2021 |
| 91 | Japan | JPN | East Asia & Pacific | High | 1980–2021 | 1960-2023 | 1991–2021 | 1980–2021 | 1991–2021 | 1980–2021 |
| 92 | Kazakhstan | KAZ | Europe & Central Asia | Upper middle | 1980–2021 | 1990-2023 | 1991–2021 | 1980–2021 | 1991–2021 | 1980–2021 |
| 93 | Kenya | KEN | Sub-Saharan Africa | Lower middle | 1980–2021 | 1960-2023 | 1991–2021 | 1980–2021 | 1991–2021 | 1980–2021 |
| 94 | Kyrgyz Republic | KGZ | Europe & Central Asia | Lower middle | 1980–2021 | 1990-2023 | 1991–2021 | 1980–2021 | 1991–2021 | 1980–2021 |
| 95 | Cambodia | KHM | East Asia & Pacific | Lower middle | 1980–2021 | 1975-2023 | 1991–2021 | 1980–2021 | 1991–2021 | 1980–2021 |
| 96 | Kiribati | KIR | East Asia & Pacific | Lower middle | 1980–2021 | 1970-2023 | not available | 1980–2021 | not available | 1980–2021 |
| 97 | St. Kitts and Nevis | KNA | Latin America & Caribbean | High | 1980–2021 | 1960-2023 | not available | 1980–2021 | not available | 1980–2021 |
| 98 | Korea, Rep. | KOR | East Asia & Pacific | High | 1980–2021 | 1960-2023 | 1991–2021 | 1980–2021 | 1991–2021 | 1980–2021 |
| 99 | Kuwait | KWT | Middle East & North Africa | High | 1980–2021 | 1962-2023 | 1991–2021 | 1980–2021 | 1991–2021 | 1980–2021 |
| 100 | Lao PDR | LAO | East Asia & Pacific | Lower middle | 1980–2021 | 1984-2023 | 1991–2021 | 1980–2021 | 1991–2021 | 1980–2021 |
| 101 | Lebanon | LBN | Middle East & North Africa | Upper middle | 1980–2021 | 1988-2022 | 1991–2021 | 1980–2021 | 1991–2021 | 1980–2021 |
| 102 | Liberia | LBR | Sub-Saharan Africa | Low | 1980–2021 | 1960-2023 | 1991–2021 | 1980–2021 | 1991–2021 | 1980–2021 |
| 103 | Libya | LBY | Middle East & North Africa | Upper middle | 1980–2021 | 1960-2023 | 1991–2021 | 1980–2021 | 1991–2021 | 1980–2021 |
| 104 | St. Lucia | LCA | Latin America & Caribbean | Upper middle | 1980–2021 | 1980-2023 | 1991–2021 | 1980–2021 | 1991–2021 | 1980–2021 |
| 105 | Sri Lanka | LKA | South Asia | Lower middle | 1980–2021 | 1960-2023 | 1991–2021 | 1980–2021 | 1991–2021 | 1980–2021 |
| 106 | Lesotho | LSO | Sub-Saharan Africa | Lower middle | 1980–2021 | 1960-2023 | 1991–2021 | 1980–2021 | 1991–2021 | 1980–2021 |
| 107 | Lithuania | LTU | Europe & Central Asia | High | 1980–2021 | 1995-2023 | 1991–2021 | 1980–2021 | 1991–2021 | 1980–2021 |
| 108 | Luxembourg | LUX | Europe & Central Asia | High | 1980–2021 | 1960-2023 | 1991–2021 | 1980–2021 | 1991–2021 | 1980–2021 |
| 109 | Latvia | LVA | Europe & Central Asia | High | 1980–2021 | 1995-2023 | 1991–2021 | 1980–2021 | 1991–2021 | 1980–2021 |
| 110 | Morocco | MAR | Middle East & North Africa | Lower middle | 1980–2021 | 1960-2023 | 1991–2021 | 1980–2021 | 1991–2021 | 1980–2021 |
| 111 | Monaco | MCO | Europe & Central Asia | High | 1980–2021 | 1970-2022 | not available | 1980–2021 | not available | not available |
| 112 | Moldova | MDA | Europe & Central Asia | Lower middle | 1980–2021 | 1990-2023 | 1991–2021 | 1980–2021 | 1991–2021 | 1980–2021 |
| 113 | Madagascar | MDG | Sub-Saharan Africa | Low | 1980–2021 | 1960-2023 | 1991–2021 | 1980–2021 | 1991–2021 | 1980–2021 |
| 114 | Maldives | MDV | South Asia | Upper middle | 1980–2021 | 1970-2023 | 1991–2021 | 1980–2021 | 1991–2021 | 1980–2021 |
| 115 | Mexico | MEX | Latin America & Caribbean | Upper middle | 1980–2021 | 1960-2023 | 1991–2021 | 1980–2021 | 1991–2021 | 1980–2021 |
| 116 | Marshall Islands | MHL | East Asia & Pacific | Upper middle | 1980–2021 | 1970-2023 | not available | 1980–2021 | not available | 1980–2021 |
| 117 | North Macedonia | MKD | Europe & Central Asia | Upper middle | 1980–2021 | 1990-2023 | 1991–2021 | 1980–2021 | 1991–2021 | 1980–2021 |
| 118 | Mali | MLI | Sub-Saharan Africa | Low | 1980–2021 | 1967-2023 | 1991–2021 | 1980–2021 | 1991–2021 | 1980–2021 |
| 119 | Malta | MLT | Middle East & North Africa | High | 1980–2021 | 1970-2023 | 1991–2021 | 1980–2021 | 1991–2021 | 1980–2021 |
| 120 | Myanmar | MMR | East Asia & Pacific | Lower middle | 1980–2021 | 1960-2023 | 1991–2021 | 1980–2021 | 1991–2021 | 1980–2021 |
| 121 | Montenegro | MNE | Europe & Central Asia | Upper middle | 1980–2021 | 1998-2023 | 1991–2021 | 1980–2021 | 1991–2021 | 1980–2021 |
| 122 | Mongolia | MNG | East Asia & Pacific | Lower middle | 1980–2021 | 1981-2023 | 1991–2021 | 1980–2021 | 1991–2021 | 1980–2021 |
| 123 | Northern Mariana Islands | MNP | East Asia & Pacific | High | 1980–2021 | 2002-2020 | not available | 1980–2021 | not available | not available |
| 124 | Mozambique | MOZ | Sub-Saharan Africa | Low | 1980–2021 | 1991-2023 | 1991–2021 | 1980–2021 | 1991–2021 | 1980–2021 |
| 125 | Mauritania | MRT | Sub-Saharan Africa | Lower middle | 1980–2021 | 1961-2023 | 1991–2021 | 1980–2021 | 1991–2021 | 1980–2021 |
| 126 | Mauritius | MUS | Sub-Saharan Africa | High | 1980–2021 | 1960-2023 | 1991–2021 | 1980–2021 | 1991–2021 | 1980–2021 |
| 127 | Malawi | MWI | Sub-Saharan Africa | Low | 1980–2021 | 1980-2023 | 1991–2021 | 1980–2021 | 1991–2021 | 1980–2021 |
| 128 | Malaysia | MYS | East Asia & Pacific | Upper middle | 1980–2021 | 1960-2023 | 1991–2021 | 1980–2021 | 1991–2021 | 1980–2021 |
| 129 | Namibia | NAM | Sub-Saharan Africa | Upper middle | 1980–2021 | 1980-2023 | 1991–2021 | 1980–2021 | 1991–2021 | 1980–2021 |
| 130 | Niger | NER | Sub-Saharan Africa | Low | 1980–2021 | 1960-2023 | 1991–2021 | 1980–2021 | 1991–2021 | 1980–2021 |
| 131 | Nigeria | NGA | Sub-Saharan Africa | Lower middle | 1980–2021 | 1960-2023 | 1991–2021 | 1980–2021 | 1991–2021 | 1980–2021 |
| 132 | Nicaragua | NIC | Latin America & Caribbean | Lower middle | 1980–2021 | 1988-2023 | 1991–2021 | 1980–2021 | 1991–2021 | 1980–2021 |
| 133 | Netherlands | NLD | Europe & Central Asia | High | 1980–2021 | 1960-2023 | 1991–2021 | 1980–2021 | 1991–2021 | 1980–2021 |
| 134 | Norway | NOR | Europe & Central Asia | High | 1980–2021 | 1960-2023 | 1991–2021 | 1980–2021 | 1991–2021 | 1980–2021 |
| 135 | Nepal | NPL | South Asia | Lower middle | 1980–2021 | 1960-2023 | 1991–2021 | 1980–2021 | 1991–2021 | 1980–2021 |
| 136 | Nauru | NRU | East Asia & Pacific | High | 1980–2021 | 1970-2023 | not available | 1980–2021 | not available | 1980–2021 |
| 137 | New Zealand | NZL | East Asia & Pacific | High | 1980–2021 | 1960-2023 | 1991–2021 | 1980–2021 | 1991–2021 | 1980–2021 |
| 138 | Oman | OMN | Middle East & North Africa | High | 1980–2021 | 1965-2023 | 1991–2021 | 1980–2021 | 1991–2021 | 1980–2021 |
| 139 | Pakistan | PAK | South Asia | Lower middle | 1980–2021 | 1960-2023 | 1991–2021 | 1980–2021 | 1991–2021 | 1980–2021 |
| 140 | Panama | PAN | Latin America & Caribbean | High | 1980–2021 | 1960-2023 | 1991–2021 | 1980–2021 | 1991–2021 | 1980–2021 |
| 141 | Peru | PER | Latin America & Caribbean | Upper middle | 1980–2021 | 1982-2023 | 1991–2021 | 1980–2021 | 1991–2021 | 1980–2021 |
| 142 | Philippines | PHL | East Asia & Pacific | Lower middle | 1980–2021 | 1960-2023 | 1991–2021 | 1980–2021 | 1991–2021 | 1980–2021 |
| 143 | Palau | PLW | East Asia & Pacific | High | 1980–2021 | 1970-2023 | not available | 1980–2021 | not available | 1990–2020 |
| 144 | Papua New Guinea | PNG | East Asia & Pacific | Lower middle | 1980–2021 | 1960-2023 | 1991–2021 | 1980–2021 | 1991–2021 | 1980–2021 |
| 145 | Poland | POL | Europe & Central Asia | High | 1980–2021 | 1990-2023 | 1991–2021 | 1980–2021 | 1991–2021 | 1980–2021 |
| 146 | Puerto Rico | PRI | Latin America & Caribbean | High | 1980–2021 | 1960-2023 | 1991–2021 | 1980–2021 | 1991–2021 | 1980–2021 |
| 147 | Portugal | PRT | Europe & Central Asia | High | 1980–2021 | 1960-2023 | 1991–2021 | 1980–2021 | 1991–2021 | 1980–2021 |
| 148 | Paraguay | PRY | Latin America & Caribbean | Upper middle | 1980–2021 | 1960-2023 | 1991–2021 | 1980–2021 | 1991–2021 | 1980–2021 |
| 149 | Qatar | QAT | Middle East & North Africa | High | 1980–2021 | 1970-2022 | 1991–2021 | 1980–2021 | 1991–2021 | 1980–2021 |
| 150 | Romania | ROU | Europe & Central Asia | High | 1980–2021 | 1987-2023 | 1991–2021 | 1980–2021 | 1991–2021 | 1980–2021 |
| 151 | Russian Federation | RUS | Europe & Central Asia | Upper middle | 1980–2021 | 1988-2023 | 1991–2021 | 1980–2021 | 1991–2021 | 1980–2021 |
| 152 | Rwanda | RWA | Sub-Saharan Africa | Low | 1980–2021 | 1960-2023 | 1991–2021 | 1980–2021 | 1991–2021 | 1980–2021 |
| 153 | Saudi Arabia | SAU | Middle East & North Africa | High | 1980–2021 | 1960-2023 | 1991–2021 | 1980–2021 | 1991–2021 | 1980–2021 |
| 154 | Sudan | SDN | Sub-Saharan Africa | Low | 1980–2021 | 1960-2023 | 1991–2021 | 1980–2021 | 1991–2021 | 1980–2021 |
| 155 | Senegal | SEN | Sub-Saharan Africa | Lower middle | 1980–2021 | 1960-2023 | 1991–2021 | 1980–2021 | 1991–2021 | 1980–2021 |
| 156 | Singapore | SGP | East Asia & Pacific | High | 1980–2021 | 1960-2023 | 1991–2021 | 1980–2021 | 1991–2021 | 1980–2021 |
| 157 | Solomon Islands | SLB | East Asia & Pacific | Lower middle | 1980–2021 | 1967-2023 | 1991–2021 | 1980–2021 | 1991–2021 | 1980–2021 |
| 158 | Sierra Leone | SLE | Sub-Saharan Africa | Low | 1980–2021 | 1960-2023 | 1991–2021 | 1980–2021 | 1991–2021 | 1980–2021 |
| 159 | El Salvador | SLV | Latin America & Caribbean | Lower middle | 1980–2021 | 1965-2023 | 1991–2021 | 1980–2021 | 1991–2021 | 1980–2021 |
| 160 | San Marino | SMR | Europe & Central Asia | High | 1980–2021 | 1999-2021 | not available | 1980–2021 | not available | 2012–2012 |
| 161 | Somalia | SOM | Sub-Saharan Africa | Low | 1980–2021 | 1960-2023 | 1991–2021 | 1980–2021 | 1991–2021 | 1980–2021 |
| 162 | Serbia | SRB | Europe & Central Asia | Upper middle | 1980–2021 | 1993-2023 | 1991–2021 | 1980–2021 | 1991–2021 | 1980–2021 |
| 163 | South Sudan | SSD | Sub-Saharan Africa | Low | 1980–2021 | 2008-2015 | 1991–2021 | 1980–2021 | 1991–2021 | 1980–2021 |
| 164 | São Tomé and Principe | STP | Sub-Saharan Africa | Lower middle | 1980–2021 | 1970-2023 | 1991–2021 | 1980–2021 | 1991–2021 | 1980–2021 |
| 165 | Suriname | SUR | Latin America & Caribbean | Upper middle | 1980–2021 | 1960-2023 | 1991–2021 | 1980–2021 | 1991–2021 | 1980–2021 |
| 166 | Slovak Republic | SVK | Europe & Central Asia | High | 1980–2021 | 1990-2023 | 1991–2021 | 1980–2021 | 1991–2021 | 1980–2021 |
| 167 | Slovenia | SVN | Europe & Central Asia | High | 1980–2021 | 1990-2023 | 1991–2021 | 1980–2021 | 1991–2021 | 1980–2021 |
| 168 | Sweden | SWE | Europe & Central Asia | High | 1980–2021 | 1960-2023 | 1991–2021 | 1980–2021 | 1991–2021 | 1980–2021 |
| 169 | Eswatini | SWZ | Sub-Saharan Africa | Lower middle | 1980–2021 | 1960-2023 | 1991–2021 | 1980–2021 | 1991–2021 | 1980–2021 |
| 170 | Seychelles | SYC | Sub-Saharan Africa | High | 1980–2021 | 1960-2023 | not available | 1980–2021 | not available | 1982–2021 |
| 171 | Syrian Arab Republic | SYR | Middle East & North Africa | Low | 1980–2021 | 1960-2021 | 1991–2021 | 1980–2021 | 1991–2021 | 1980–2021 |
| 172 | Chad | TCD | Sub-Saharan Africa | Low | 1980–2021 | 1960-2023 | 1991–2021 | 1980–2021 | 1991–2021 | 1980–2021 |
| 173 | Togo | TGO | Sub-Saharan Africa | Low | 1980–2021 | 1960-2023 | 1991–2021 | 1980–2021 | 1991–2021 | 1980–2021 |
| 174 | Thailand | THA | East Asia & Pacific | Upper middle | 1980–2021 | 1960-2023 | 1991–2021 | 1980–2021 | 1991–2021 | 1980–2021 |
| 175 | Tajikistan | TJK | Europe & Central Asia | Low | 1980–2021 | 1990-2023 | 1991–2021 | 1980–2021 | 1991–2021 | 1980–2021 |
| 176 | Turkmenistan | TKM | Europe & Central Asia | Upper middle | 1980–2021 | 1987-2023 | 1991–2021 | 1980–2021 | 1991–2021 | 1980–2021 |
| 177 | Timor-Leste | TLS | East Asia & Pacific | Lower middle | 1980–2021 | 1990-2023 | 1991–2021 | 1980–2021 | 1991–2021 | 1980–2021 |
| 178 | Tonga | TON | East Asia & Pacific | Upper middle | 1980–2021 | 1975-2022 | 1991–2021 | 1980–2021 | 1991–2021 | 1980–2021 |
| 179 | Trinidad and Tobago | TTO | Latin America & Caribbean | High | 1980–2021 | 1960-2023 | 1991–2021 | 1980–2021 | 1991–2021 | 1980–2021 |
| 180 | Tunisia | TUN | Middle East & North Africa | Lower middle | 1980–2021 | 1961-2023 | 1991–2021 | 1980–2021 | 1991–2021 | 1980–2021 |
| 181 | Turkey | TUR | Europe & Central Asia | Upper middle | 1980–2021 | 1960-2023 | 1991–2021 | 1980–2021 | 1991–2021 | 1980–2021 |
| 182 | Tuvalu | TUV | East Asia & Pacific | Upper middle | 1980–2021 | 1970-2023 | not available | 1980–2021 | not available | 1980–2021 |
| 183 | Tanzania | TZA | Sub-Saharan Africa | Lower middle | 1980–2021 | 1960-2023 | 1991–2021 | 1980–2021 | 1991–2021 | 1980–2021 |
| 184 | Uganda | UGA | Sub-Saharan Africa | Low | 1980–2021 | 1960-2023 | 1991–2021 | 1980–2021 | 1991–2021 | 1980–2021 |
| 185 | Ukraine | UKR | Europe & Central Asia | Lower middle | 1980–2021 | 1987-2023 | 1991–2021 | 1980–2021 | 1991–2021 | 1980–2021 |
| 186 | Uruguay | URY | Latin America & Caribbean | High | 1980–2021 | 1960-2023 | 1991–2021 | 1980–2021 | 1991–2021 | 1980–2021 |
| 187 | United States | USA | North America | High | 1980–2021 | 1960-2023 | 1991–2021 | 1980–2021 | 1991–2021 | 1980–2021 |
| 188 | Uzbekistan | UZB | Europe & Central Asia | Lower middle | 1980–2021 | 1987-2023 | 1991–2021 | 1980–2021 | 1991–2021 | 1980–2021 |
| 189 | St. Vincent and the Grenadines | VCT | Latin America & Caribbean | Upper middle | 1980–2021 | 1960-2023 | 1991–2021 | 1980–2021 | 1991–2021 | 1980–2021 |
| 190 | Venezuela, RB | VEN | Latin America & Caribbean | Upper middle | 1980–2021 | 1960-2014 | 1991–2021 | 1980–2021 | 1991–2021 | 1980–2021 |
| 191 | Virgin Islands (U.S.) | VIR | Latin America & Caribbean | High | 1980–2021 | 2002-2021 | 1991–2021 | 1980–2021 | 1991–2021 | 1980–2021 |
| 192 | Vietnam | VNM | East Asia & Pacific | Lower middle | 1980–2021 | 1985-2023 | 1991–2021 | 1980–2021 | 1991–2021 | 1980–2021 |
| 193 | Vanuatu | VUT | East Asia & Pacific | Lower middle | 1980–2021 | 1979-2023 | 1991–2021 | 1980–2021 | 1991–2021 | 1980–2021 |
| 194 | Samoa | WSM | East Asia & Pacific | Upper middle | 1980–2021 | 1970-2023 | 1991–2021 | 1980–2021 | 1991–2021 | 1980–2021 |
| 195 | Yemen, Rep. | YEM | Middle East & North Africa | Low | 1980–2021 | 1990-2023 | 1991–2021 | 1980–2021 | 1991–2021 | 1980–2021 |
| 196 | South Africa | ZAF | Sub-Saharan Africa | Upper middle | 1980–2021 | 1960-2023 | 1991–2021 | 1980–2021 | 1991–2021 | 1980–2021 |
| 197 | Zambia | ZMB | Sub-Saharan Africa | Lower middle | 1980–2021 | 1960-2023 | 1991–2021 | 1980–2021 | 1991–2021 | 1980–2021 |
| 198 | Zimbabwe | ZWE | Sub-Saharan Africa | Lower middle | 1980–2021 | 1960-2023 | 1991–2021 | 1980–2021 | 1991–2021 | 1980–2021 |

# S2 Table. Associations between the average GDPpc growth in different time window (years) and age-standardised suicide rate

| The average  % change of  GDPpc in  earlier N Years | The difference in age-standardized suicide rate per 100,000 people according to 1 % growth of GDPpc during the specific period | | |
| --- | --- | --- | --- |
|  | Total | Male | Female |
|  | β (95% CI)^b^ | β (95% CI)^b^ | β (95% CI)^b^ |
| 1 | 0.0007 (-0.003 to 0.0044) | 0.0012 (-0.005 to 0.0075) | 0.0005 (-0.0014 to 0.0023) |
| 2 | -0.0001 (-0.0053 to 0.0051) | -0.0002 (-0.0089 to 0.0084) | 0.0005 (-0.0022 to 0.0031) |
| 3 | -0.0019 (-0.0081 to 0.0043) | -0.003 (-0.0134 to 0.0074) | -0.0004 (-0.0036 to 0.0028) |
| 4 | -0.0052 (-0.0123 to 0.0019) | -0.0083 (-0.0202 to 0.0036) | -0.002 (-0.0057 to 0.0016) |
| 5 | -0.0095 (-0.0175 to -0.0014)* | -0.0147 (-0.0282 to -0.0012)* | -0.0045 (-0.0087 to -0.0003)* |
| 6 | -0.0115 (-0.0203 to -0.0028)* | -0.0174 (-0.032 to -0.0027)* | -0.0061 (-0.0107 to -0.0015)* |
| 7 | -0.0128 (-0.0223 to -0.0033)* | -0.0181 (-0.0339 to -0.0023)* | -0.0078 (-0.0128 to -0.0028)* |
| 8 | -0.0135 (-0.0238 to -0.0032)* | -0.0173 (-0.0344 to -0.0002)* | -0.0097 (-0.0152 to -0.0043)** |
| 9 | -0.0148 (-0.026 to -0.0036)* | -0.017 (-0.0355 to 0.0016) | -0.0123 (-0.0182 to -0.0063)** |
| 10 | -0.0171 (-0.0293 to -0.0049)* | -0.0178 (-0.0379 to 0.0023) | -0.0156 (-0.0221 to -0.009)** |
| 11 | -0.0203 (-0.0335 to -0.0071)* | -0.0199 (-0.0417 to 0.0018) | -0.0194 (-0.0265 to -0.0123)** |
| 12 | -0.0236 (-0.0378 to -0.0094)* | -0.0217 (-0.0451 to 0.0016) | -0.0235 (-0.0312 to -0.0158)** |
| 13 | -0.0265 (-0.0416 to -0.0113)** | -0.0224 (-0.0473 to 0.0024) | -0.0279 (-0.0362 to -0.0196)** |
| 14 | -0.0327 (-0.0489 to -0.0165)** | -0.0282 (-0.0546 to -0.0018)* | -0.0339 (-0.0428 to -0.025)** |
| 15 | -0.0396 (-0.0568 to -0.0223)** | -0.0353 (-0.0632 to -0.0073)* | -0.0399 (-0.0495 to -0.0304)** |
| 16 | -0.0461 (-0.0644 to -0.0278)** | -0.0412 (-0.0708 to -0.0116)* | -0.0464 (-0.0567 to -0.0362)** |
| 17 | -0.0525 (-0.0718 to -0.0331)** | -0.0478 (-0.0791 to -0.0166)* | -0.0522 (-0.0631 to -0.0413)** |
| 18 | -0.0589 (-0.0792 to -0.0385)** | -0.0551 (-0.0878 to -0.0223)** | -0.0573 (-0.0688 to -0.0458)** |
| 19 | -0.0646 (-0.0858 to -0.0433)** | -0.0616 (-0.0957 to -0.0275)** | -0.0615 (-0.0736 to -0.0494)** |
| 20 | -0.0703 (-0.0925 to -0.0481)** | -0.068 (-0.1036 to -0.0325)** | -0.0656 (-0.0783 to -0.0528)** |

^a^The number of years used to calculate the average growth of GDPpc from the year when the suicide rate was recorded.

^b^Point estimates and 95% confidence intervals show the difference in suicide rate among countries having 1% growth of GDPpc during the specific period. For example, in ‘Both sexes’, point estimates and 95% confidence intervals shows how an average growth of GDPpc in ‘3’ years are associated with the suicide rate of the last year of the ‘3’ years. All models were adjusted for annual GDPpc (US$), unemployment as a proportion of the total workforce (%), proportion of the population aged over 65 years (%), and fertility rate per female in the population.

* p < 0.05

GDPpc, gross domestic product per capita; CI, confidence interval

# S3 Table. Diagnostics and number of observations for the associations between the average of absolute values of % change of GDPpc in different time window (years) and age-standardised suicide rate.

| **The average  % change of  GDPpc in  earlier N Years** | **Total** | | | | **Male** | | | | **Female** | | | |
| --- | --- | --- | --- | --- | --- | --- | --- | --- | --- | --- | --- | --- |
|  | **Number of observations** | **Degrees of freedom** | **R squared** | **F value** | **Number of observations** | **Degrees of freedom** | **R squared** | **F value** | **Number of observations** | **Degrees of freedom** | **R squared** | **F value** |
| 1 | 5468 | 5252 | 0.115 | 113.9 | 5468 | 5252 | 0.117 | 116.1 | 5468 | 5252 | 0.093 | 89.8 |
| 2 | 5438 | 5222 | 0.111 | 109.2 | 5438 | 5222 | 0.114 | 112.0 | 5438 | 5222 | 0.089 | 84.6 |
| 3 | 5407 | 5191 | 0.108 | 104.9 | 5407 | 5191 | 0.111 | 108.3 | 5407 | 5191 | 0.084 | 79.7 |
| 4 | 5373 | 5157 | 0.105 | 101.3 | 5373 | 5157 | 0.109 | 105.2 | 5373 | 5157 | 0.081 | 75.6 |
| 5 | 5335 | 5119 | 0.103 | 98.0 | 5335 | 5119 | 0.107 | 102.0 | 5335 | 5119 | 0.078 | 72.1 |
| 6 | 5296 | 5080 | 0.099 | 93.3 | 5296 | 5080 | 0.103 | 97.5 | 5296 | 5080 | 0.075 | 68.4 |
| 7 | 5256 | 5040 | 0.095 | 88.2 | 5256 | 5040 | 0.099 | 92.2 | 5256 | 5040 | 0.072 | 65.0 |
| 8 | 5214 | 4999 | 0.091 | 83.7 | 5214 | 4999 | 0.095 | 87.4 | 5214 | 4999 | 0.070 | 62.5 |
| 9 | 5172 | 4957 | 0.088 | 80.0 | 5172 | 4957 | 0.092 | 83.2 | 5172 | 4957 | 0.069 | 60.8 |
| 10 | 5129 | 4914 | 0.087 | 77.6 | 5129 | 4914 | 0.089 | 80.2 | 5129 | 4914 | 0.069 | 60.4 |
| 11 | 5085 | 4870 | 0.085 | 75.1 | 5085 | 4870 | 0.087 | 76.9 | 5085 | 4870 | 0.069 | 60.4 |
| 12 | 5035 | 4820 | 0.082 | 72.1 | 5035 | 4820 | 0.083 | 73.0 | 5035 | 4820 | 0.070 | 60.4 |
| 13 | 4984 | 4769 | 0.080 | 69.6 | 4984 | 4769 | 0.081 | 69.8 | 4984 | 4769 | 0.071 | 60.5 |
| 14 | 4933 | 4718 | 0.080 | 67.9 | 4933 | 4718 | 0.079 | 67.4 | 4933 | 4718 | 0.072 | 61.4 |
| 15 | 4882 | 4667 | 0.079 | 66.3 | 4882 | 4667 | 0.077 | 65.0 | 4882 | 4667 | 0.074 | 62.3 |
| 16 | 4831 | 4616 | 0.077 | 64.5 | 4831 | 4616 | 0.075 | 62.3 | 4831 | 4616 | 0.076 | 63.4 |
| 17 | 4777 | 4562 | 0.077 | 63.1 | 4777 | 4562 | 0.074 | 60.3 | 4777 | 4562 | 0.077 | 63.8 |
| 18 | 4723 | 4508 | 0.077 | 62.3 | 4723 | 4508 | 0.073 | 59.1 | 4723 | 4508 | 0.079 | 64.0 |
| 19 | 4669 | 4454 | 0.077 | 61.6 | 4669 | 4454 | 0.073 | 58.2 | 4669 | 4454 | 0.079 | 63.8 |
| 20 | 4615 | 4403 | 0.077 | 61.2 | 4615 | 4403 | 0.073 | 57.4 | 4615 | 4403 | 0.080 | 63.5 |

# S4 Table. Associations between the average GDPpc growth in different time window (years) and age-standardised suicide rate

| The average  % change of  GDPpc in  earlier N Years^a^ | **The difference in age-standardized suicide rate per 100,000 people according to 1 % growth of GDPpc during the specific period β (95% CI)^b^** | | | | | | | | |  |
| --- | --- | --- | --- | --- | --- | --- | --- | --- | --- | --- |
|  | **High Income** | | | **Upper Middle Income** | | | **Lower Middle and Lower Income** | | |  |
|  | Total | Male | Female | Total | Male | Female | Total | Male | Female | |
| 1 | -0.0009  (-0.0141 to 0.0123) | -0.0007  (-0.0227 to 0.0212) | -0.001  (-0.0075 to 0.0055) | 0.0026  (-0.003 to 0.0081) | 0.0042  (-0.0056 to 0.0141) | 0.0014  (-0.0015 to 0.0043) | -0.0012  (-0.0064 to 0.004) | -0.0023  (-0.0108 to 0.0062) | -0.0002  (-0.0028 to 0.0024) | |
| 2 | 0.0083  (-0.0088 to 0.0254) | 0.0132  (-0.0151 to 0.0415) | 0.0041  (-0.0044 to 0.0126) | 0.0025  (-0.005 to 0.01) | 0.0038  (-0.0095 to 0.0171) | 0.0021  (-0.0018 to 0.0061) | -0.0055  (-0.0133 to 0.0023) | -0.0101  (-0.0228 to 0.0027) | -0.0017  (-0.0056 to 0.0022) | |
| 3 | 0.019  (-0.0019 to 0.04) | 0.0314  (-0.0032 to 0.0659) | 0.0083  (-0.0023 to 0.0188) | 0.0024  (-0.0066 to 0.0113) | 0.004  (-0.0118 to 0.0199) | 0.0019  (-0.0028 to 0.0066) | -0.0102  (-0.0198 to -0.0006)* | -0.0182  (-0.0339 to -0.0025)* | -0.0034  (-0.0082 to 0.0014) | |
| 4 | 0.0268  (0.0028 to 0.0509)* | 0.0457  (0.0062 to 0.0852)* | 0.0097  (-0.0026 to 0.022) | 0.0013  (-0.009 to 0.0116) | 0.0029  (-0.0155 to 0.0212) | 0.0012  (-0.0043 to 0.0067) | -0.0167  (-0.0279 to -0.0055)* | -0.0296  (-0.0479 to -0.0112)* | -0.0056  (-0.0112 to 0)* | |
| 5 | 0.0325  (0.0057 to 0.0593)* | 0.0556  (0.0119 to 0.0993)* | 0.0109  (-0.0029 to 0.0247) | 0.0013  (-0.0108 to 0.0133) | 0.0042  (-0.0172 to 0.0256) | 0.0001  (-0.0063 to 0.0064) | -0.0256  (-0.0383 to -0.0129)** | -0.0451  (-0.0659 to -0.0244)** | -0.0086  (-0.0149 to -0.0022)* | |
| 6 | 0.0358  (0.0076 to 0.0639)* | 0.0581  (0.0124 to 0.1038)* | 0.0138  (-0.0009 to 0.0285) | 0.0027  (-0.0105 to 0.016) | 0.0085  (-0.0151 to 0.032) | -0.0008  (-0.0078 to 0.0063) | -0.0333  (-0.0474 to -0.0191)** | -0.0586  (-0.0816 to -0.0356)** | -0.0108  (-0.0178 to -0.0037)* | |
| 7 | 0.0386  (0.009 to 0.0682)* | 0.0608  (0.0129 to 0.1086)* | 0.0159  (0.0002 to 0.0315)* | 0.0045  (-0.0101 to 0.0191) | 0.0134  (-0.0124 to 0.0392) | -0.0014  (-0.0093 to 0.0064) | -0.0392  (-0.055 to -0.0234)** | -0.0686  (-0.0943 to -0.0429)** | -0.0125  (-0.0204 to -0.0046)* | |
| 8 | 0.0397  (0.0084 to 0.071)* | 0.063  (0.0125 to 0.1134)* | 0.015  (-0.0017 to 0.0317) | 0.0082  (-0.0078 to 0.0241) | 0.0221  (-0.006 to 0.0501) | -0.0018  (-0.0105 to 0.0069) | -0.0438  (-0.0613 to -0.0262)** | -0.0756  (-0.1041 to -0.0471)** | -0.0141  (-0.0229 to -0.0054)* | |
| 9 | 0.0425  (0.0097 to 0.0753)* | 0.0704  (0.0178 to 0.1231)* | 0.0129  (-0.0048 to 0.0305) | 0.0108  (-0.0068 to 0.0284) | 0.0291  (-0.0018 to 0.0601) | -0.0025  (-0.0122 to 0.0072) | -0.0482  (-0.0677 to -0.0287)** | -0.0821  (-0.1137 to -0.0505)** | -0.0158  (-0.0255 to -0.006)* | |
| 10 | 0.0442  (0.0096 to 0.0787)* | 0.0733  (0.0179 to 0.1286)* | 0.013  (-0.0057 to 0.0317) | 0.012  (-0.0076 to 0.0316) | 0.0345  (0.0001 to 0.0689)* | -0.0042  (-0.015 to 0.0066) | -0.0522  (-0.0736 to -0.0307)** | -0.0866  (-0.1214 to -0.0519)** | -0.018  (-0.0288 to -0.0072)* | |
| 11 | 0.0434  (0.0067 to 0.0801)* | 0.0729  (0.0142 to 0.1315)* | 0.0113  (-0.0087 to 0.0313) | 0.0108  (-0.0106 to 0.0321) | 0.0354  (-0.0019 to 0.0728) | -0.0068  (-0.0186 to 0.005) | -0.0547  (-0.0784 to -0.0311)** | -0.0889  (-0.1271 to -0.0507)** | -0.0197  (-0.0316 to -0.0077)* | |
| 12 | 0.0406  (0.0016 to 0.0796)* | 0.0724  (0.0102 to 0.1345)* | 0.0062  (-0.0152 to 0.0276) | 0.008  (-0.0148 to 0.0309) | 0.0338  (-0.0061 to 0.0736) | -0.01  (-0.0228 to 0.0028) | -0.0574  (-0.0835 to -0.0314)** | -0.0914  (-0.1334 to -0.0494)** | -0.021  (-0.0341 to -0.0078)* | |
| 13 | 0.0265  (-0.0151 to 0.0681) | 0.0544  (-0.0118 to 0.1206) | -0.0049  (-0.0278 to 0.0181) | 0.0062  (-0.0179 to 0.0302) | 0.034  (-0.0077 to 0.0756) | -0.0136  (-0.0272 to 0.0001) | -0.0569  (-0.0853 to -0.0285)** | -0.0886  (-0.1343 to -0.0429)** | -0.0205  (-0.0348 to -0.0061)* | |
| 14 | 0.0044  (-0.0402 to 0.0491) | 0.0212  (-0.0497 to 0.0921) | -0.0168  (-0.0415 to 0.0079) | -0.0012  (-0.0264 to 0.024) | 0.0256  (-0.0178 to 0.0691) | -0.02  (-0.0346 to -0.0054)* | -0.0575  (-0.0883 to -0.0268)** | -0.0863  (-0.1357 to -0.0369)** | -0.0212  (-0.0368 to -0.0056)* | |
| 15 | -0.0212  (-0.0695 to 0.0271) | -0.0201  (-0.0967 to 0.0566) | -0.0281  (-0.0549 to -0.0013)* | -0.0126  (-0.0389 to 0.0136) | 0.0105  (-0.0345 to 0.0555) | -0.0281  (-0.0437 to -0.0125)** | -0.0563  (-0.0893 to -0.0233)** | -0.0814  (-0.1344 to -0.0284)* | -0.021  (-0.0377 to -0.0042)* | |
| 16 | -0.0462  (-0.0983 to 0.006) | -0.0587  (-0.1413 to 0.024) | -0.0401  (-0.069 to -0.0111)* | -0.0264  (-0.0538 to 0.001) | -0.0071  (-0.0538 to 0.0396) | -0.0387  (-0.0552 to -0.0221)** | -0.0524  (-0.0877 to -0.0171)* | -0.0723  (-0.1289 to -0.0157)* | -0.0194  (-0.0373 to -0.0014)* | |
| 17 | -0.0722  (-0.1274 to -0.017)* | -0.1045  (-0.1919 to -0.0172)* | -0.0475  (-0.0782 to -0.0168)* | -0.0423  (-0.0708 to -0.0138)* | -0.0288  (-0.077 to 0.0194) | -0.05  (-0.0677 to -0.0323)** | -0.0453  (-0.0829 to -0.0076)* | -0.057  (-0.1172 to 0.0033) | -0.0168  (-0.0359 to 0.0023) | |
| 18 | -0.097  (-0.1535 to -0.0406)** | -0.1488  (-0.2381 to -0.0595)* | -0.0532  (-0.0846 to -0.0218)** | -0.0582  (-0.0875 to -0.0289)** | -0.05  (-0.0993 to -0.0006)* | -0.0619  (-0.0807 to -0.0431)** | -0.0395  (-0.0796 to 0.0005) | -0.0442  (-0.1083 to 0.0199) | -0.0144  (-0.0347 to 0.0059) | |
| 19 | -0.1134  (-0.1704 to -0.0564)** | -0.1801  (-0.2701 to -0.0902)** | -0.0538  (-0.0857 to -0.0219)** | -0.0689  (-0.0992 to -0.0387)** | -0.0611  (-0.1118 to -0.0103)* | -0.0728  (-0.0927 to -0.0529)** | -0.0376  (-0.0801 to 0.0049) | -0.0392  (-0.1071 to 0.0288) | -0.0122  (-0.0337 to 0.0093) | |
| 20 | -0.1145  (-0.1721 to -0.057)** | -0.1876  (-0.2783 to -0.0969)** | -0.0466  (-0.079 to -0.0142)* | -0.0809  (-0.112 to -0.0498)** | -0.0734  (-0.1253 to -0.0216)* | -0.085  (-0.1059 to -0.064)** | -0.0397  (-0.0846 to 0.0052) | -0.0402  (-0.1119 to 0.0316) | -0.0116  (-0.0344 to 0.0111) | |

^a^The number of years used to calculate the average growth of GDPpc from the year when the suicide rate was recorded.

^b^Point estimates and 95% confidence intervals show the difference in suicide rate among countries having 1% growth of GDPpc during the specific period. For example, in ‘Both sexes’, point estimates and 95% confidence intervals shows how an average growth of GDPpc in ‘3’ years are associated with the suicide rate of the last year of the ‘3’ years.

Model 1: adjusted for annual GDPpc (US$), proportion of the population aged over 65 years (%), and fertility rate per female in the population / Model 2: Model 1 + unemployment as a proportion of the total workforce (%), expenditures for health care per capita (US $), and female employment (%) labor market

* p < 0.05

GDPpc, gross domestic product per capita; CI, confidence interval

# S5 Table. Diagnostics and number of observations for the associations between the average GDPpc growth in different time window (years) and age-standardised suicide rate

| **Group** | **The average  % change of  GDPpc in  earlier N Years** | **Total** | | | | **Male** | | | | **Female** | | | |
| --- | --- | --- | --- | --- | --- | --- | --- | --- | --- | --- | --- | --- | --- |
|  |  | **Number of observations** | **Degrees of freedom** | **R squared** | **F value** | **Number of observations** | **Degrees of freedom** | **R squared** | **F value** | **Number of observations** | **Degrees of freedom** | **R squared** | **F value** |
| ***High income*** | 1 | 1668 | 1577 | 0.104 | 30.5 | 1668 | 1577 | 0.116 | 34.4 | 1668 | 1577 | 0.074 | 20.9 |
|  | 2 | 1658 | 1567 | 0.101 | 29.3 | 1658 | 1567 | 0.112 | 33.1 | 1658 | 1567 | 0.071 | 20.0 |
|  | 3 | 1648 | 1557 | 0.098 | 28.3 | 1648 | 1557 | 0.110 | 32.0 | 1648 | 1557 | 0.068 | 19.0 |
|  | 4 | 1638 | 1547 | 0.097 | 27.6 | 1638 | 1547 | 0.108 | 31.2 | 1638 | 1547 | 0.066 | 18.3 |
|  | 5 | 1627 | 1536 | 0.096 | 27.2 | 1627 | 1536 | 0.108 | 30.9 | 1627 | 1536 | 0.064 | 17.6 |
|  | 6 | 1616 | 1525 | 0.095 | 26.6 | 1616 | 1525 | 0.106 | 30.2 | 1616 | 1525 | 0.063 | 17.1 |
|  | 7 | 1605 | 1514 | 0.093 | 25.9 | 1605 | 1514 | 0.104 | 29.3 | 1605 | 1514 | 0.062 | 16.6 |
|  | 8 | 1594 | 1503 | 0.091 | 25.0 | 1594 | 1503 | 0.102 | 28.3 | 1594 | 1503 | 0.060 | 15.9 |
|  | 9 | 1583 | 1492 | 0.089 | 24.3 | 1583 | 1492 | 0.100 | 27.5 | 1583 | 1492 | 0.058 | 15.3 |
|  | 10 | 1572 | 1481 | 0.088 | 23.9 | 1572 | 1481 | 0.099 | 27.1 | 1572 | 1481 | 0.057 | 15.0 |
|  | 11 | 1561 | 1470 | 0.087 | 23.2 | 1561 | 1470 | 0.097 | 26.4 | 1561 | 1470 | 0.056 | 14.4 |
|  | 12 | 1550 | 1459 | 0.086 | 22.7 | 1550 | 1459 | 0.097 | 26.0 | 1550 | 1459 | 0.055 | 14.0 |
|  | 13 | 1539 | 1448 | 0.084 | 22.1 | 1539 | 1448 | 0.095 | 25.4 | 1539 | 1448 | 0.054 | 13.8 |
|  | 14 | 1528 | 1437 | 0.083 | 21.7 | 1528 | 1437 | 0.094 | 24.9 | 1528 | 1437 | 0.055 | 13.8 |
|  | 15 | 1517 | 1426 | 0.082 | 21.2 | 1517 | 1426 | 0.092 | 24.2 | 1517 | 1426 | 0.056 | 14.1 |
|  | 16 | 1506 | 1415 | 0.081 | 20.7 | 1506 | 1415 | 0.090 | 23.2 | 1506 | 1415 | 0.057 | 14.4 |
|  | 17 | 1494 | 1403 | 0.080 | 20.4 | 1494 | 1403 | 0.089 | 22.8 | 1494 | 1403 | 0.057 | 14.2 |
|  | 18 | 1482 | 1391 | 0.082 | 20.6 | 1482 | 1391 | 0.090 | 22.9 | 1482 | 1391 | 0.058 | 14.4 |
|  | 19 | 1470 | 1379 | 0.083 | 20.9 | 1470 | 1379 | 0.092 | 23.3 | 1470 | 1379 | 0.058 | 14.1 |
|  | 20 | 1458 | 1369 | 0.083 | 20.6 | 1458 | 1369 | 0.092 | 23.2 | 1458 | 1369 | 0.056 | 13.5 |
| ***Upper-middle income*** | 1 | 1527 | 1441 | 0.225 | 69.9 | 1527 | 1441 | 0.203 | 61.3 | 1527 | 1441 | 0.232 | 72.4 |
|  | 2 | 1518 | 1432 | 0.232 | 72.0 | 1518 | 1432 | 0.212 | 64.1 | 1518 | 1432 | 0.234 | 73.0 |
|  | 3 | 1508 | 1422 | 0.237 | 73.6 | 1508 | 1422 | 0.219 | 66.4 | 1508 | 1422 | 0.235 | 72.9 |
|  | 4 | 1496 | 1410 | 0.243 | 75.2 | 1496 | 1410 | 0.225 | 68.1 | 1496 | 1410 | 0.238 | 73.5 |
|  | 5 | 1483 | 1397 | 0.243 | 74.9 | 1483 | 1397 | 0.226 | 67.8 | 1483 | 1397 | 0.240 | 73.5 |
|  | 6 | 1470 | 1384 | 0.235 | 70.8 | 1470 | 1384 | 0.218 | 64.1 | 1470 | 1384 | 0.237 | 71.6 |
|  | 7 | 1457 | 1371 | 0.225 | 66.4 | 1457 | 1371 | 0.209 | 60.2 | 1457 | 1371 | 0.232 | 69.1 |
|  | 8 | 1443 | 1357 | 0.216 | 62.2 | 1443 | 1357 | 0.200 | 56.5 | 1443 | 1357 | 0.229 | 67.1 |
|  | 9 | 1428 | 1342 | 0.209 | 59.2 | 1428 | 1342 | 0.193 | 53.3 | 1428 | 1342 | 0.231 | 67.0 |
|  | 10 | 1412 | 1326 | 0.206 | 57.5 | 1412 | 1326 | 0.188 | 51.1 | 1412 | 1326 | 0.235 | 68.0 |
|  | 11 | 1396 | 1310 | 0.201 | 54.9 | 1396 | 1310 | 0.180 | 47.9 | 1396 | 1310 | 0.240 | 69.1 |
|  | 12 | 1377 | 1291 | 0.192 | 51.0 | 1377 | 1291 | 0.169 | 43.7 | 1377 | 1291 | 0.244 | 69.6 |
|  | 13 | 1358 | 1272 | 0.182 | 47.2 | 1358 | 1272 | 0.158 | 39.7 | 1358 | 1272 | 0.247 | 69.6 |
|  | 14 | 1339 | 1253 | 0.172 | 43.3 | 1339 | 1253 | 0.146 | 35.6 | 1339 | 1253 | 0.249 | 69.3 |
|  | 15 | 1320 | 1234 | 0.162 | 39.7 | 1320 | 1234 | 0.133 | 31.6 | 1320 | 1234 | 0.251 | 68.9 |
|  | 16 | 1301 | 1215 | 0.155 | 37.0 | 1301 | 1215 | 0.123 | 28.4 | 1301 | 1215 | 0.255 | 69.2 |
|  | 17 | 1281 | 1195 | 0.151 | 35.5 | 1281 | 1195 | 0.116 | 26.1 | 1281 | 1195 | 0.260 | 69.9 |
|  | 18 | 1261 | 1175 | 0.149 | 34.4 | 1261 | 1175 | 0.110 | 24.2 | 1261 | 1175 | 0.263 | 70.0 |
|  | 19 | 1241 | 1155 | 0.146 | 33.0 | 1241 | 1155 | 0.104 | 22.2 | 1241 | 1155 | 0.268 | 70.4 |
|  | 20 | 1221 | 1135 | 0.147 | 32.5 | 1221 | 1135 | 0.100 | 21.1 | 1221 | 1135 | 0.274 | 71.6 |
| ***Lower-middle and low income*** | 1 | 2273 | 2162 | 0.079 | 30.8 | 2273 | 2162 | 0.066 | 25.3 | 2273 | 2162 | 0.116 | 47.2 |
|  | 2 | 2262 | 2151 | 0.080 | 31.2 | 2262 | 2151 | 0.067 | 25.8 | 2262 | 2151 | 0.116 | 47.2 |
|  | 3 | 2251 | 2140 | 0.082 | 31.8 | 2251 | 2140 | 0.069 | 26.6 | 2251 | 2140 | 0.117 | 47.3 |
|  | 4 | 2239 | 2128 | 0.085 | 32.8 | 2239 | 2128 | 0.073 | 27.8 | 2239 | 2128 | 0.119 | 47.8 |
|  | 5 | 2225 | 2114 | 0.090 | 34.8 | 2225 | 2114 | 0.078 | 30.0 | 2225 | 2114 | 0.121 | 48.5 |
|  | 6 | 2210 | 2099 | 0.094 | 36.2 | 2210 | 2099 | 0.083 | 31.6 | 2210 | 2099 | 0.123 | 49.1 |
|  | 7 | 2194 | 2083 | 0.095 | 36.4 | 2194 | 2083 | 0.084 | 31.8 | 2194 | 2083 | 0.123 | 48.7 |
|  | 8 | 2177 | 2067 | 0.095 | 36.3 | 2177 | 2067 | 0.084 | 31.5 | 2177 | 2067 | 0.123 | 48.2 |
|  | 9 | 2161 | 2051 | 0.095 | 35.9 | 2161 | 2051 | 0.083 | 31.0 | 2161 | 2051 | 0.122 | 47.5 |
|  | 10 | 2145 | 2035 | 0.095 | 35.5 | 2145 | 2035 | 0.082 | 30.1 | 2145 | 2035 | 0.122 | 47.2 |
|  | 11 | 2128 | 2018 | 0.094 | 34.8 | 2128 | 2018 | 0.079 | 29.0 | 2128 | 2018 | 0.122 | 46.9 |
|  | 12 | 2108 | 1998 | 0.093 | 34.2 | 2108 | 1998 | 0.077 | 27.9 | 2108 | 1998 | 0.123 | 46.5 |
|  | 13 | 2087 | 1977 | 0.092 | 33.2 | 2087 | 1977 | 0.074 | 26.4 | 2087 | 1977 | 0.123 | 46.0 |
|  | 14 | 2066 | 1956 | 0.090 | 32.3 | 2066 | 1956 | 0.071 | 25.0 | 2066 | 1956 | 0.123 | 45.6 |
|  | 15 | 2045 | 1935 | 0.090 | 31.7 | 2045 | 1935 | 0.069 | 24.1 | 2045 | 1935 | 0.124 | 45.8 |
|  | 16 | 2024 | 1914 | 0.089 | 31.3 | 2024 | 1914 | 0.068 | 23.4 | 2024 | 1914 | 0.126 | 45.9 |
|  | 17 | 2002 | 1892 | 0.090 | 31.1 | 2002 | 1892 | 0.068 | 22.9 | 2002 | 1892 | 0.128 | 46.1 |
|  | 18 | 1980 | 1870 | 0.091 | 31.1 | 1980 | 1870 | 0.068 | 22.7 | 1980 | 1870 | 0.130 | 46.5 |
|  | 19 | 1958 | 1848 | 0.093 | 31.4 | 1958 | 1848 | 0.070 | 23.1 | 1958 | 1848 | 0.132 | 46.8 |
|  | 20 | 1936 | 1827 | 0.094 | 31.6 | 1936 | 1827 | 0.071 | 23.2 | 1936 | 1827 | 0.133 | 46.7 |

**S1 Fig. World Map of Countries by Income Group**

The map was generated with data from Natural Earth (http://www.naturalearthdata.com/) using the R package "rnaturalearth" (v0.3.2) (https://github.com/ropensci/rnaturalearth)

**S2 Fig.** **Association between the average percentage change in GDPpc in different time periods (years) and age-standardized suicide rate by countries according to income in various model specifications.**

**Model 1:** GDP growth only

**Model 2:** GDP growth + Unemployment rate

**Model 3:** GDP growth + Unemployment rate + Population aged 65

**Model 4:** GDP growth + Unemployment rate + Population aged 65 + Female labor force

participation rate

**Model 5: GDP growth + Unemployment rate + Population aged 65+ + Female labor force participation rate + fertility rate**
